# Ellagic Acid Triggers the Necrosis of Differentiated Human Enterocytes Exposed to 3-Nitro-Tyrosine: An MS-Based Proteomic Study

**DOI:** 10.3390/antiox11122485

**Published:** 2022-12-17

**Authors:** Silvia Díaz-Velasco, Josué Delgado, Fernando J. Peña, Mario Estévez

**Affiliations:** 1 Food Technology and Quality (TECAL), Institute of Meat and Meat Products (IPROCAR), Universidad de Extremadura, 10003 Cáceres, Spain; 2 Food Hygiene and Safety (HISEALI), Institute of Meat and Meat Products (IPROCAR), Universidad de Extremadura, 10003 Cáceres, Spain; 3 Spermatology Laboratory, Universidad de Extremadura, 10003 Cáceres, Spain

**Keywords:** 3-Nitro-L-tyrosine, ellagic acid, proteomics, flow cytometry, protein oxidation, MHC class II, mitochondria

## Abstract

To study the molecular basis of the toxicological effect of a dietary nitrosated amino acid, namely, 3-nitrotyrosine (3-NT), differentiated human enterocytes were exposed to dietary concentrations of this species (200 μM) and analyzed for flow cytometry, protein oxidation markers and MS-based proteomics. The possible protective role of a dietary phytochemical, ellagic acid (EA) (200 μM), was also tested. The results revealed that cell viability was significantly affected by exposure to 3-NT, with a concomitant significant increase in necrosis (*p* < 0.05). 3-NT affected several biological processes, such as histocompatibility complex class II (MHC class II), and pathways related to type 3 metabotropic glutamate receptors binding. Addition of EA to 3-NT-treated cells stimulated the toxicological effects of the latter by reducing the abundance of proteins involved in mitochondrial conformation. These results emphasize the impact of dietary nitrosated amino acids in intestinal cell physiology and warn about the potential negative effects of ellagic acid when combined with noxious metabolites.

## 1. Introduction

Oxidative stress is related to assorted health disorders such as type 2 diabetes mellitus, hypertension, atherosclerosis, pulmonary diseases, systemic inflammatory response syndrome, aging, cancer and Alzheimer’s disease, among others [1,2]. Reactive oxygen species (ROS) and reactive nitrogen species (RNS) are involved in oxidative stress through the imbalance between ROS/RNS and antioxidant defenses [2]. Specifically, 3-nitro-L-tyrosine (3-NT), a nitrosation product of tyrosine, is formed during processing of nitrite-added foods (cured foods) [3,4] and is recognized as an indicator of protein nitration in foods and other biological systems [5]. In addition, 3-NT has been used as a biomarker of acute and chronic inflammatory processes, which indicates the involvement of the nitrosation product in pathological conditions [6,7]. While the potential toxicity of 3-NT has been documented, the underlying molecular mechanisms are poorly understood. Blanchard-Fillion et al. [7] reported that 3-NT is transported into rat neuronal cells via the L-aromatic amino acid transporter and is mis-incorporated into proteins such as α-tubulin and decreases the synthesis of dopamine. In these cells, 3-NT is eventually able to induce caspase-3 mediated apoptosis which the authors associated with the onset of neurological disorders such as Parkinson’s disease [7]. Consistently, Zhang and Wei [8] found that 3-NT induced apoptosis in cardiomyocytes in a type 2 diabetes rat model. Neutralization of 3-NT counteracted the apoptosis and diabetic myocardiopathy in rats, which reasonably incriminates the chemical species in the pathogenesis [8]. In a previous in vitro study [9], we reported that 3-NT caused oxidative stress, accretion of oxidized proteins and necrotic events in human enterocytes, leading to a significant decrease in viability of these cells. 3-NT has been found in concentrations of up to 200 μM in food protein suspensions and in fermented sausages [5,10,11]. Interestingly, processed meats, including cured meats, were identified as group 1 carcinogens by the International Agency for Research on Cancer [12] based on epidemiological studies. It is wholly unknown whether nitrosated proteins and amino acids, such as 3-NT, are contributing or not to the potential health concerns of dietary cured meats on the gastrointestinal tract (GIT). 

Ellagic acid (EA) is a polyphenolic compound that is widely found in plant materials such as pomegranate fruit [13]. The protective effect of EA against ROS and oxidative stress has been documented in both in vitro and in vivo studies [14]. Much less is known about the potential protection that EA and other phytochemicals may exert against RNS and nitrosative stress. We hypothesized whether EA could counteract the noxious effects of 3-NT in a biological system. Advanced methodologies, such as label-free quantitative MS-based proteomics, along with other techniques such as flow cytometry, have been recently found to provide original insight into the molecular mechanisms of the toxicological effects of oxidized amino acids [15]. 

Both chemical species under study (3-NT and EA) are common dietary components and both are firstly exposed to cells from the intestinal epithelium. The purpose of this study was to decipher the molecular basis of the potential toxicological effect of 3-NT and the hypothetical protective role of EA on differentiated human colon CACO-2 cells. These cells were used as an experimental model of human enterocytes upon spontaneous differentiation for 21 days [16]. To achieve the aforementioned objective, these human intestinal cells were treated with food-compatible doses of 3-NT and EA (200 μM) for 72 h, and subsequently analyzed by flow cytometry analyses, oxidative stress and high-resolution mass-spectrometry-based proteomics using a Q-Exactive Orbitrap MS/MS. 

## 2. Materials and Methods

### 2.1. Cells and Chemicals

Human colon adenocarcinoma CACO-2 cell line was purchased from ECACC (European Collection of Authenticated Cell Cultures, Salisbury, UK). 3-NT (CAS Number 621-44-3) and EA (CAS Number 476-66-4) were acquired from Sigma-Aldrich (Sigma-Aldrich, Steinheim, Germany). The other reagents were obtained from Panreac (Panreac Química, S. A., Barcelona, Spain), Sigma-Aldrich (Sigma-Aldrich, Steinheim, Germany) and Fisher (Fisher Scientific S.L., Madrid, Spain). Water used was purified by passage through a Milli-Q system (Millipore Corp., Bedford, MA, USA).

### 2.2. Cell Culture

Passages between 40 and 46 of human colon adenocarcinoma CACO-2 cell line were used. Cells were cultured in T-75 flasks at 37 ºC in a humidified incubator 95% air/5% CO_2_ in Eagle’s Minimum Essential Medium (EMEM) supplemented with fetal bovine serum (FBS) (10% *v/v*), non-essential amino acids (1% *v/v*) and L-glutamine (1% *v/v*). Cells were allowed to differentiate into enterocytes for 21 days upon reaching 90% confluence. Finally, cells were collected using a combination of 0.25% trypsin and 1 mM EDTA.

### 2.3. Experimental Setting

Both 3-NT and EA were dissolved in 10 mL of the culture medium (supplemented EMEM) at a final concentration of 200 μM in. The concentration of 3-NT used in the present study is within the range found in processed foods and food digests [5,10]. The same concentration of EA was used to study the possible protective effect at equivalent concentration. 3-NT and 3-NT+EA were then added and exposed to differentiated CACO-2 cells in T-75 flasks at 37 °C in a humidified incubator 95%air/5%CO_2_. For comparative purposes, CONTROL cells were incubated under equivalent conditions in standard EMEM. The entire assay was repeated five times (true replicates) in independent flasks for 3-NT, 3-NT+EA and CONTROL cells. After 72 h, cells were harvested using 0.25% trypsin in 1 mM EDTA, centrifuged for 5 min at 126 g and suspended in 1 mL of phosphate-buffered saline (PBS) solution for further analysis. The sampling time was chosen in accordance with previous preliminary studies, aiming to set condition parameters to guarantee a clear visualization of proteome changes in response to exposure to the chemical species under study. 

### 2.4. Flow Cytometry Analyses

Flow cytometry analyses were conducted using a Cytoflex® LX flow cytometer (Beckman Coulter, Brea, CA, USA) equipped with red, blue, violet, ultraviolet, yellow and infrared lasers. The instrument was calibrated daily using specific calibration beads provided by the manufacturer. A compensation overlap was performed before each experiment. Files were exported as FCS files and analyzed using FlowJoV 10.4.1 Software (Ashland, OR, USA). Samples were diluted in PBS to a final concentration 5 × 10^6^ cells/mL and stained with 1 µL of Hoechst 33,342 (16.2 mM stock solution), 1 µL of CellROX (2.5 mM stock solution) and 1 µL CellEvent Caspase-3/7 Green Detection Reagent (2 mM stock solution) to measure cell viability, ROS occurrence and apoptosis events, respectively. After thorough mixing, the cell suspension was incubated at room temperature for 20 min in the dark, then was loaded with 1 µL ethidium homodimer (1.167 mM in DMSO) and incubated for a further 10 min to measure necrosis events. Finally, the samples were filtered through MACS® smartStrainer 30 µm filters and immediately run on the flow cytometer. The controls consisted of unstained and single-stained controls to properly set gates and compensations.

### 2.5. Quantification of Protein Carbonyls

Both α-aminoadipic semialdehyde (α-AS) and γ-glutamic semialdehyde (γ-GS), two main protein carbonyls, were quantified in differentiated human enterocytes. The procedure reported by Utrera et al. [17] was replicated with slight changes described as follows. Briefly, 100 µL of the cell lysates were treated with 1 mL of cold 10% trichloroacetic acid (TCA) solution, and then proteins were precipitated with centrifugation at 2240× *g* for 5 min at 4 °C. The supernatant was removed, and the resulting pellet was treated with 1 mL of cold 5% TCA. Proteins were then precipitated by applying cold 5% TCA and centrifugation at 2240× *g* for 5 min. Protein-bound carbonyls were then derivatized with p-amino-benzoic acid (ABA) followed by a subsequent acid hydrolysis in 6M HCl and 110 °C for 18 h. The hydrolysates were evaporated at 40 °C in a vacuum concentrator, and the generated residues were reconstituted with 200 μL of Milli-Q water and then filtered through hydrophilic polypropylene GH Polypro (GHP) syringe filters (0.45 μm pore size, Pall Corporation, NJ, USA) for HPLC analysis. Details on the chromatography apparatus, as well as on the separation, elution and identification of the compounds of interest, were published in Utrera et al. [17]. Standard α-AS-ABA and γ-GS-ABA were synthesized according to the protocol described by Akagawa et al. [18]. Identification of α-AS and γ-GS in samples was carried out by comparing their retention times with those of the synthesized standard compounds. A standard curve of ABA was prepared by injecting increasing known concentrations of this fluorescent species (0.1 to 0.5 mM). The peaks corresponding to both α-AS-ABA and γ-GS-ABA were manually integrated and the resulting areas plotted against the ABA standard curve. Results are expressed as nmol of protein carbonyl per mg of protein.

### 2.6. Endogenous Antioxidant Enzymes

The activity of two endogenous antioxidant enzymes, namely, catalase (CAT) and superoxide dismutase (SOD), was determined by spectrophotometric methods. One unit (U) of catalase was defined as the amount of protein from cell lysate needed to decompose 1 mmol of H_2_O_2_ per min. One unit of SOD was defined as the amount of protein from cell lysate required to inhibit pyrogallol autoxidation by 50%.

### 2.7. Sample Preparation for LC-MS/MS Based Proteomics

The procedure carried out for the preparation of tryptic peptides from cultured cells has already been described by Díaz-Velasco et al. [15]. Samples were treated with 0.5 mL of lysis buffer pH 7.5 (100 mM Tris-HCl, 50 mM NaCl, 10% glycerol, 0.5 M EDTA pH 8.5) containing 100 mM PMSF (Phenylmethansulfonylfluorid) and 100 μg/mL Pepstatin in a 1:100 proportion. Samples were stirred and sonicated (Branson Ultrasonics, Danbury, CT, USA). Lysates were incubated on ice for 1 h and centrifuged at 9032× *g* for 10 min at 4 °C. Supernatants were analyzed for protein concentration. Aliquots containing 50 µg of proteins were partially run in SDS-PAGE (4% stacking and 12% separating). Runs were stopped as soon as samples reached the separating part of the gel to be digested in-gel. The gel was stained with Coomassie blue R250 and each lane was cut into 1 mm^3^ pieces and subjected to in-gel digestion. Samples were incubated with 0.5 M DTT in 50 mM ammonium bicarbonate for 20 min at 56 °C for protein reduction. The resulting free thiol (-SH) groups were alkylated by incubating the samples with 0.55 M iodoacetamide in 50 mM ammonium bicarbonate for 15 min in the dark at room temperature (22 °C). Afterwards, samples were treated with 50 mM ammonium bicarbonate, ProteaseMAX (Promega, Madison, WI, USA) and trypsin (Promega, USA) and incubated for 2 h at 37 °C. The proteolysis was stopped by adding formic acid. Supernatants were dried in vacuo and subsequently reconstituted with loading buffer (98% milli-Q water, 2% acetonitrile and 0.05% trifluoroacetic acid). 

### 2.8. Label-Free Quantitative Proteomic Analyses

A Q-Exactive Plus mass spectrometer coupled to a Dionex Ultimate 3000 RSLCnano (Thermo Scientific, Waltham, MA, USA) analyzed 5 μg from each digest. Data was collected using a Top15 method for MS/MS scans following the procedure described by Delgado et al. [19], with some modifications as described in [15]. Comparative proteome abundance and data analysis were conducted using MaxQuant software (version 1.6.0.15.0) and Perseus (v 1.6.14.0) to organize the data and perform statistical analysis. Carbamidomethylation of cysteines was set as a fixed modification; oxidation of methionines and acetylation of N-terminals were set as variable modifications. Database searching was carried out against Homo sapiens UNIPROT protein database. The maximum peptide/protein false discovery rates (FDR) were set to 1% based on comparison to a reverse database. The MaxLFQ algorithm was used to generate normalized spectral intensities and infer relative protein abundance. Proteins were only retained in final analysis if they were detected in at least two replicates from at least one treatment, and the proteins that matched to a contaminant database or the reverse database were removed. Quantitative analysis was performed using a T-test to compare treatments with the control. Fold change (FC) is expressed as Log_2_. This refers to the degree of quantity change for a particular protein between cells (control vs. treatment). If FC is <1, the change denotes a decrease in the concentration of protein in treated samples (vs. control) while fold change >1 indicates a significant increase in the concentration of that protein in the treated sample (vs. control). When a given protein is only present in one of the groups, fold change cannot be measured and such condition is denoted in tables as “C” (if protein is only present in C, in other words, exposure to whatever treatment leads to a complete depletion of the protein) or 3-NT (if protein is only present in 3-NT-exposed cells) or 3-NT+EA (if protein is only present in cells exposed to the combination of both species). A qualitative analysis was also performed to detect proteins that were found in at least three replicates of a given group of samples (i.e., treated) and were undetectable in the counterpart group (i.e., control). Proteins satisfying one of these two aforementioned criteria were identified as discriminating proteins, and their corresponding genes were grouped by biological processes and molecular functions using ClueGO (v. 2.5.6) [20]. To define term–term inter-relations and functional groups based on shared genes between the terms, the Kappa score was established at 0.4. Three GO terms and 4% of genes covered were set as the minimum required to be retained in the final result. The *p*-value was corrected by Bonferroni step down and set as *p* < 0.05. 

### 2.9. Statistical Analysis

All experiments were carried out five times and each individual sample was measured twice for flow cytometry. Data was analyzed for normality and homoscedasticity, and the effect of the exposure to 3-NT and 3-NT+EA was evaluated by Analysis of Variance (ANOVA). Tukey’s test was used for multiple comparisons of the means. The effect of the incubation time on the same measurements was assessed by Student’s t-test. SPSS (version 15.0) was used for statistical analysis of the data and the significance level was set at *p* < 0.05.

## 3. Results

### 3.1. Flow Cytometry and Protein Oxidation Markers

**Figure 1:**
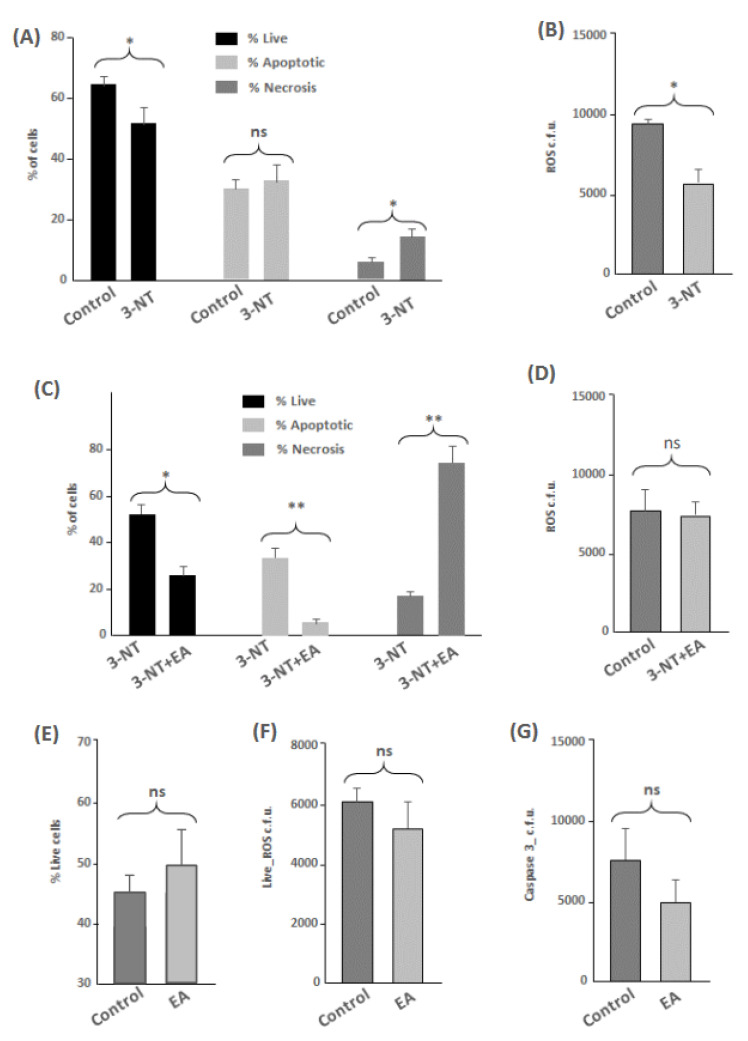
Percentages of live cells (Hoechst +), apoptotic cells (Caspase 3+) and necrotic cells (Ethidium homodimer +) on human enterocytes as affected by exposure to 200 μM 3-NT for 72 h compared to control counterparts (**A**). Relative fluorescence units (r.f.u.) of ROS occurrence (Cell Rox +) on human enterocytes as affected by exposure to 200 μM 3-NT for 72 h compared to control counterparts (**B**). Percentages of live cells (Hoechst +), apoptotic cells (Caspase 3+) and necrotic cells (Ethidium homodimer +) on differentiated human enterocytes as affected by exposure to 200 μM 3-NT+ 200 μM EA for 72 h compared to 200 μM 3-NT treated cells (**C**). Relative fluorescence units (r.f.u.) of ROS occurrence (Cell Rox +) on human enterocytes as affected by exposure to 200 μM 3-NT+ 200 μM EA for 72 h compared to control counterparts (**D**). Percentages of live cells (Hoechst +) (**E**), relative fluorescence units (r.f.u.) of ROS occurrence (Cell Rox +) (F) and relative fluorescence units (r.f.u.) of apoptotic events (Caspase 3+) (**G**) on human enterocytes as affected by exposure to 200 μM EA for 72 h compared to control counterparts. Results are expressed as means ± standard deviations. Asterisk on top of bars denote significant differences (* *p* < 0.05; ** *p* < 0.01) between paired group of samples (control vs. 3-NT; 3-NT vs. 3-NT). Ns: no significant differences.

In this study, the parameters analyzed by flow cytometry were cell viability, ROS occurrence, apoptosis and necrosis. The results showed a significant decrease in the percentage of live cells among differentiated human enterocytes exposed to 3-NT compared to control cells (*p* < 0.05) (Figure 1A). The decreased viability of cells exposed to 3-NT was manifested in a significant increase of necrotic events (*p* < 0.05) (Figure 1A). Cells exposed to 3-NT showed a significant decrease in ROS occurrence (*p* < 0.05) (Figure 1B) and no significant effect was observed in the percentage of live cells for caspase-3-mediated apoptotic events (Figure 1A) as compared to control counterparts. Cells exposed to the combination of 3-NT+EA showed a significant decrease in the percentage of live cells (*p* < 0.05) (Figure 1C), a significant reduction in the percentage of live cells of apoptotic events (*p* < 0.01) and a significant and remarkable increase in necrotic events (*p* < 0.01), as compared to cells exposed only to 3-NT. No significant differences were found in ROS occurrence between non-treated cells and 3-NT+EA (Figure 1D). To elucidate whether the effects observed in the 3-NT+EA group were a result of the occurrence of EA or the interaction between both species, an additional assay was performed to assess the impact of EA alone. This phytochemical showed a mild (non-significant) positive effect on human enterocytes as compared with a control group, with a clear trend to an increased viability and decreased apoptotic and necrotic events (Figure 1E–G). 

**Figure 2:**
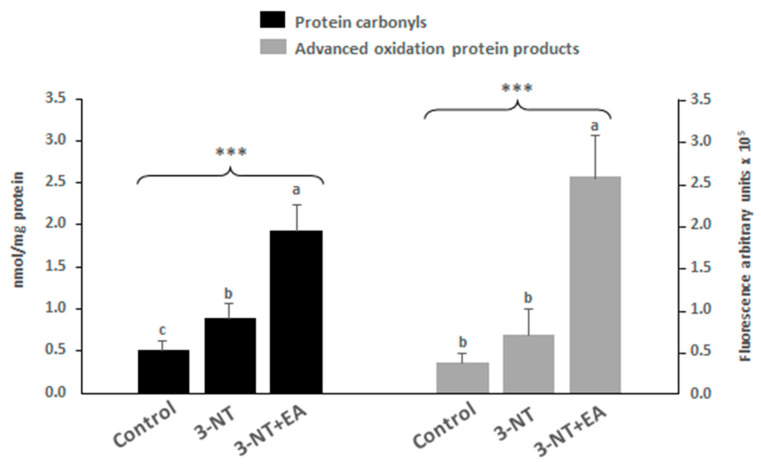
Protein carbonyls (nmol/mg protein of α-AS and γ-GS) (black bars) and advanced oxidation protein products (Pentosidine; fluorescence intensity) (grey bars) on differentiated human enterocytes upon exposure to 200 μM 3-NT and 200 μM 3-NT+200 μM EA for 72 h. Asterisks on top of bars denote significant differences between group of samples in ANOVA (*** *p* < 0.001). (a–c) Different letters on top of bars denote significant differences between means in post-hoc Tukey tests (*p* < 0.05).

The analysis of protein oxidation markers α-AS and γ-GS was assessed by the detection of early (specific protein carbonyls, α-AS and γ-GS) and advanced oxidation protein products (AOPPs) (Figure 2). The results show that the exposure to 3-NT led to a significant accretion of protein carbonyls in enterocytes, while this increase was not significant for AOPPs. The addition of EA to 3-NT-treated cells caused another significant and highly remarkable increase in protein carbonyls and AOPPs as compared to both Control and 3-NT counterparts.

### 3.2. Proteomic Analyses

The MS-based proteomic platform enabled the identification of 2398 proteins in total. All these proteins were identified with at least two peptides and a FDR <1%. Quantitative (*p* < 0.05) and qualitative (only detected in one condition) changes in protein abundance were studied (Tables S1 and S2). To analyze the possible harmful effects of 3-NT, a first comparison was made between the proteomes from non-treated control cells versus 3-NT-treated cells. In this case, 150 proteins were significantly influenced by 3-NT, with 56 among those being found in lower abundance in cells treated with 3-NT, and 15 being only found in non-treated control samples. On the other hand, 63 proteins were detected in higher quantity in cells exposed to 3-NT and 16 were only found in 3-NT-treated cells. To study the hypothetical protective role of EA against 3-NT, an additional comparison was made between the proteomes from 3-NT-treated cells versus 3-NT+EA-treated cells. In this case, 1214 proteins were significantly influenced by 3-NT+EA; among those, 481 were detected in lower abundance in cells exposed to 3-NT+EA, 365 were found in higher quantity in 3-NT+EA treated cells, 23 were only detected in the presence of 3-NT+EA and 345 were only found in 3-NT treated cells. For a systematized and comprehensible description and discussion of results, discriminating proteins were grouped by biological processes and molecular functions. The comparison between non-treated control cells versus 3-NT-treated cells (possible harmful effect of 3-NT) is shown in Graphs SG1A-SG1D in the Supplementary Material. The comparison between 3-NT-treated cells versus 3-NT+EA-treated cells (3-NT vs. hypothetical protective role of EA) is shown in Graphs SG2A-SG2D in the Supplementary Material. Specific terms for each of these processes and full details of discriminating proteins and associated genes are provided in Supplementary Material Tables S3–S10. Table 1 and Table 2 show a selection of representative proteins from each relevant biological process and molecular function affected by the presence of 3-NT and 3-NT+EA. Only discriminating proteins having a defined biological significance are presented in the following sections. 

#### 3.2.1. Proteins Found in Lower Relative Quantity in Cells Exposed to 3-NT Compared to Control Counterparts

##### Antigen Processing and Presentation of Exogenous Peptide Antigen via MHC Class II 

Among all biological processes affected by the exposure of 3-NT, the proteins implicated in the antigen processing and presentation of exogenous peptide antigen via MHC class II (SG. 1A) were particularly targeted (63.64%, *p* < 0.01). The proteins involved in this process (Supplementary Table S3) were beta-centractin (ACTR1B; fold change: 0.87), AP-1 complex subunit sigma-1A (AP1S1, fold change: 0.71), cytoplasmic dynein 1 heavy chain 1 (DYNC1H1; fold change: 0.53), myosin-10 (MYH10; fold change: 0.68) and F-actin-capping protein subunit beta (CAPZB, only in control) (Table 1 and Tables S1–S3).

##### Cytoplasmic Translational Initiation 

The exposure of 3-NT caused a significant decrease (18.18%, *p* < 0.01) (SG. 1A) in proteins involved in the cytoplasmic translational initiation. Namely, eukaryotic translation initiation factor 2 subunit 3 (EIF2S3; fold change: 0.86), eukaryotic translation initiation factor 2 subunit 3B (EIF2S3B; fold change: 0.86) and eukaryotic translation initiation factor 3 subunit D (EIF3D; fold change: 0.75) were particularly affected by 3-NT exposure (Table 1 and Tables S1–S3).

##### Nucleosome Assembly

Other biological processes affected by the exposure of 3-NT (9.09%, *p* < 0.01) (SG. 1A) were related to the nucleosome assembly. The histones H2B type 1-L (H2BC13), H2B type 1-M (H2BC14), H2B type 1-N (H2BC15), H2B type 2-F (H2BC18), H2B type 1-D (H2BC5) and H2B type 1-H (H2BC9), all with a fold change of 0.73, were found in lower quantities in 3-NT-treated cells as compared to the control counterparts. The member 5 of subfamily A of SWI/SNF-related matrix-associated actin-dependent regulator of chromatin (SMARCA5, fold change: 0.78) also appeared in this process in a lower amount along with the aforementioned histones (Table 1 and Tables S1–S3).

##### Mitotic Cytokinesis

Mitotic cytokinesis was also affected by 3-NT exposure (9.09%, *p* < 0.01) (SG. 1A). Related proteins found in lower quantities in 3-NT cells compared to control cells were MYH10, Rho-associated protein kinase 2 (ROCK2, only in control), sorting nexin-9 (SNX9, fold change 0.75) and the STAM-binding protein (STAMBP; only in control) (Table 1 and Tables S1–S3). 

##### Intramolecular Oxidoreductase Activity, Transposing C=C bonds

Within molecular functions, the most affected process by the exposure of 3-NT was the intramolecular oxidoreductase activity, transposing C=C bonds (50.0%, *p* < 0.01) (SG. 1B). Among the most affected proteins, we identified the D-dopachrome decarboxylase (DDT; fold change: 0.74), D-dopachrome decarboxylase-like protein (DDTL; fold change: 0.74), 3 beta-hydroxysteroid dehydrogenase/Delta 5→4-isomerase type 1 (HSD3B1; only in control) and 3 beta-hydroxysteroid dehydrogenase/Delta 5→4-isomerase type 2 (HSD3B2; only in control) (Table 1 and Tables S1–S4).

##### Tau Protein Binding

Proteins involved in the tau protein binding activity were also diminished in cells exposed to 3-NT (25.0%, *p* < 0.01) (SG. 1B), such as the subunit 1 of dynactin (DCTN1; fold change: 0.82), glycogen synthase kinase-3 beta (GSK3B, only in control) and Rho-associated protein kinase 2 (ROCK2; only in control) (Table 1 and Tables S1–S4).

##### Other Proteins of Biological Significance Found in Lower Relative Quantity in 3-NT-Treated Cells

Other biologically relevant proteins found in lower quantities were delta (24)-sterol reductase (DHCR24, fold change: 0.55), interferon regulatory factor 3 (IRF3, fold change: 0.63), lipopolysaccharide-responsive and beige-like anchor protein (LRBA, fold change: 0.70), subunit RPB2 of DNA-directed RNA polymerase II (POLR2B, fold change: 0.71), protein 1 of ribosome-binding (RRBP1, fold change: 0.78), subunit alpha of coatomer (COPA, fold change: 0.79) and leukocyte elastase inhibitor (SERPINB1, fold change: 0.87) (Table 1 and Table S1). Some other proteins were only found in control samples (completely suppressed in the presence of 3-NT). Among the latter, we emphasize Arf-GAP with Rho-GAP domain, ANK repeat and PH domain-containing protein 1 (ARAP1), subunit RPB1 of DNA-directed RNA polymerase II (POLR2A), component 5 of exocyst complex (EXOC5), alcohol dehydrogenase 4 (ADH4), protein 2 of developmentally regulated GTP-binding (DRG2), STAM-binding protein (STAMBP), Cirhin (CIRH1A), coronin-2A (CORO2A), U6 snRNA-associated Sm-like protein LSm2 (LSM2) and factor 1 of TRPM8 channel-associated (TCAF1) (Table 1 and Table S1).

#### 3.2.2. Proteins Found in Higher Relative Quantity in Cells Exposed to 3-NT Compared to Control Counterparts

##### Positive Regulation of Cyclic-Nucleotide Phosphodiesterase Activity

The biological process most widely affected by the exposure of 3-NT (87.01%, *p* < 0.01) was positive regulation of cyclic nucleotide phosphodiesterase activity (SG. 1C). Among the most affected pathways, we identified the nitric oxide synthase regulator activity, adenylate cyclase activator activity and positive and negative regulation of ryanodine-sensitive calcium-release channel activity (Supplementary Table S5). The proteins affected in all these processes were calmodulin-1, calmodulin-2 and calmodulin-3 (CALM1, CALM2 and CALM3; Fold change: 1.29) (Table 1 and Tables S1–S5).

##### Type 3 Metabotropic Glutamate Receptor Binding

The most affected molecular function in the presence of 3-NT was type 3 metabotropic glutamate receptor binding (86.36%, *p* < 0.05) (SG. 1D) in which the aforementioned calmodulin proteins, are implicated (CALM1, CALM2 and CALM3) (Supplementary Table S6). Other pathways affected were nitric oxide synthase regulator activity, adenylate cyclase activator activity and N-terminal myristoylation domain binding (Supplementary Table S6).

##### Other Proteins of Biological Significance Found in Higher Relative Quantity in 3-NT-Treated Cells

Other biologically relevant proteins found in significantly higher quantity in 3-NT-treated cells were: neutral amino acid transporter B(0) (SLC1A5, fold change: 2.22), serine/arginine repetitive matrix protein 1 (SRRM1, fold change: 2.13), tumor susceptibility gene 101 protein (TSG101, fold change: 2.00), Cadherin-17 (CDH17, fold change: 1.71), 60S ribosomal protein L11 (RPL11, fold change: 1.57) and mitochondrial glutamate carrier 1 (SLC25A22, fold change: 1.30) (Table 1 and Table S1).

Proteins of biological significance only found in 3-NT-treated cells that have not been included previously but are complementary to previous results were: U1 small nuclear ribonucleoprotein C (SNRPC), CD2 antigen cytoplasmic tail-binding protein 2 (CD2BP2), carcinoembryonic antigen-related cell adhesion molecule 1 (CEACAM1), La-related protein 4B (LARP4B), lipid droplet-regulating VLDL assembly factor AUP1 (AUP1), non-homologous end-joining factor 1 (NHEJ1), subunit RPB3 of DNA-directed RNA polymerase II (POLR2C) and proton-coupled folate transporter (SLC46A1) (Table 1 and Table S1).

#### 3.2.3. Proteins Found in Lower Relative Quantity in Cells Exposed to 3-NT+EA Compared to 3-NT Counterparts

##### Intracellular Transport

The biological process most influenced by the exposure of 3-NT+EA (18.72%, p<0.01) was intracellular transport (SG. 2A). The cytoplasmic translational initiation process was one of the most affected in proteins such as subunit alpha of translation initiation factor eIF-2B (EIF2B1, only in 3-NT), subunit 2 of eukaryotic translation initiation factor 2(EIF2S2, only in 3-NT), eukaryotic initiation factor 4A-II (EIF4A2, fold change: 0.43), eukaryotic translation initiation factor 4E (EIF4E, fold change: 0.46), RNA-binding protein 4 (RBM4, fold change: 0.73) and monocarboxylate transporter 1 (SLC16A1, fold change: 0.40), among others (Table 2 and Tables S2–S7). These proteins are also implicated in other processes such as translational initiation, protein targeting to ER (endoplasmic reticulum) translation regulator activity and nucleic acid binding (Supplementary Table S7), which were, for this reason, also affected by 3-NT+AE exposure. Ribosomal proteins were also found in lower abundance such as La-related protein 1 (LARP1, only in 3-NT), 60S ribosomal protein L12 (RPL12, fold change: 0.27), 60S ribosomal protein L23a (RPL23A, fold change: 0.26), 60S ribosomal protein L13 (RPL13, fold change: 0.22), 40S ribosomal protein S7 (RPS7, fold change: 0.33) and 60S ribosomal protein L11 (RPL11, fold change: 0.49) (Table 2 and Tables S2–S7).

##### Purine Nucleotide Metabolic Process

Proteins involved in the metabolic process of purine nucleotides were found at lower quantity in 3-NT+EA treated cells compared to 3-NT samples (8.15%, *p* < 0.01) (SG. 2A). Specific routes such as the cristae formation, mitochondrial ATP synthesis coupled proton transport and energy coupled proton transport, down electrochemical gradient, were severely decreased. Some relevant proteins in the aforementioned processes are the subunits alpha (ATP5F1A), beta (ATP5F1B) and gamma (ATP5F1C) of the mitochondrial ATP synthase (fold changes: 0.17; 0.31 and 0.50, respectively). The subunit delta of the same mitochondrial ATP synthase, (ATP5F1D) was not found in enterocytes when EA was combined to 3-NT. Other relevant mitochondrial proteins were also found in lower abundance such as the subunit B1 of the mitochondrial ATP synthase F(0) complex (ATP5PB, fold change: 0.50), the subunit d of the mitochondrial ATP synthase, (ATP5PD, fold change: 0.46), the subunit O of the mitochondrial ATP synthase, (ATP5PO, fold change: 0.42), the subunit 5B of the mitochondrial cytochrome c oxidase, (COX5B, fold change: 0.61) and the mitochondrial stomatin-like protein 2 (STOML2, fold change: 0.38) (Table 2 and Tables S2–S7). Other remarkable proteins implicated in the ATP biosynthetic process, such as pyruvate kinase PKM (PKM, fold change: 0.70) and calcium-binding mitochondrial carrier protein Aralar2 (SLC25A13, fold change: 0.74), were in lower abundance in cells exposed to 3-NT+EA than in those exposed only to 3-NT. Interestingly, various relevant proteins involved in the mitochondrial membrane organization were also remarkably found in lower quantities when EA was combined with 3-NT. Among the latter, we may emphasize AFG3-like protein 2 (AFG3L2, fold change: 0.50), mitochondrial dynamin-like 120 kDa protein (OPA1, fold change, 0.34), the subunits MIC19 (CHCHD3), MIC60 (IMMT) and MIC13 (MICOS13) of the mitochondrial MICOS complex, (fold changes: 0.07; 0.47, respectively, while the latter was only present in 3-NT-treated cells), DnaJ homolog protein (member 11, subfamily C) (DNAJC11, fold change: 0.33), the mitochondrial EF-hand domain-containing protein 1 (LETM1, fold change: 0.40), the mitochondrial inner membrane protein OXA1L (OXA1L, only in 3-NT) and sorting and assembly machinery component 50 homolog (SAMM50, fold change: 0.60) (Table 2 and Tables S2–S7).

##### Nucleotide Binding and Nitric Oxide Synthase Regulator Activity

Among molecular functions in human enterocytes, nucleotide binding was the most affected by 3-NT+EA exposure (26.83%, *p* < 0.01), along with nitric oxide synthase regulator activity (7.32%, *p* < 0.01) (SG. 2B). In nucleotide binding, terms related to this molecular function such as ATPase activity, GTP binding, nucleotide binding and ATP binding were influenced by the exposure of 3-NT+EA (Supplementary Table S8). Proteins in lower abundance were mitochondrial adenylate kinase 4 (AK4, fold change: 0.38), mitochondrial ATP-binding cassette (member 10, sub-family B), (ABCB10, only in 3-NT), dynamin-2 (DNM2, fold change: 0.26), mitochondrial ribosome-releasing factor 2 (GMF2, only in 3-NT), histone H1.4 (H1-4, fold change: 0.08), calmodulin-1 and calmodulin-3 (CALM1, CALM3 fold change: 0.73), among many others (Table 2 and Tables S2–S8).

Within the processes implicated in the regulating activity of nitric oxide synthase, the type 3 metabotropic glutamate receptor binding, the N-terminal myristoylation domain binding, the adenylate cyclase activator activity and the nitric oxide synthase regulator activity were some of the most affected in human enterocytes by exposure to 3-NT+EA. Proteins found in lower quantity were CALM1, CALM2, CALM3, subunit beta of calcium/calmodulin-dependent protein kinase type II (CAMK2B, fold change: 0.66), phosphatidylinositol-binding clathrin assembly protein (PICALM, only in 3-NT), subunit alpha of guanine nucleotide-binding protein G(s) isoforms XLas (GNAS, fold change: 0.67), ATPase 4 of plasma membrane calcium-transporting (ATP2B4, only in 3-NT), epidermal growth factor receptor (EGFR, only in 3-NT), heat shock protein HSP 90-alpha (HSP90AA1, fold change: 0.82) and heat shock protein HSP 90-beta (HSP90AB1, fold change: 0.68) (Table 2 and Tables S2–S8). 

#### 3.2.4. Proteins Found in Higher Relative Quantity in Cells Exposed to 3-NT+EA Compared to 3-NT Counterparts

##### Posttranscriptional Regulation of Gene Expression

The exposure to 3-NT+EA affected the post-transcriptional regulation of gene expression (19.28%, *p* < 0.01) in human enterocytes (SG. 2C). Within this biological process, the more affected functions were peptidase activity and proteasomal ubiquitin-independent protein catabolic process. In this regard, several subunits of the proteasome system were found in higher abundance, namely, alpha type-1 (PSMA1, fold change: 1.18), alpha type-6 (PSMA6, fold change: 1.62), beta type-1 (PSMB1, fold change: 1.40), beta type-2 (PSMB2, fold change: 1.79), beta type-3 (PSMB3, fold change: 1.55), beta type-4 (PSMB4, fold change: 1.32) and beta type-5 (PSMB5, fold change: 1.19) (Table 2 and Tables S2–S9).

##### Organic Substance Catabolic Process

The catabolism of organic substances was also upregulated (10.78%, *p* < 0.01) by the influence of 3-NT+EA (SG. 2C). Proteins found in higher quantity in the catabolic process of L-serine were the iron–sulfur subunit of the mitochondrial succinate dehydrogenase [ubiquinone], (SDHB, fold change: 1.87), both the cytosolic (SHMT1) and mitochondrial (SHMT2) serine hydroxymethyltransferases, (fold changes: 1.74 and 1.34, respectively) and sorbitol dehydrogenase (SORD, fold change: 1.46) (Table 2 and Tables S2–S9). Other relevant enzymes such as catalase (CAT, fold change: 2.66) and mitochondrial glycine dehydrogenase (decarboxylating) (GLDC, fold change: 2.23) were identified as some of the most affected by the exposure to 3-NT+EA (Table 2 and Tables S2–S9).

##### Protein-Containing Complex Assembly

The protein-containing complex assembly was also one of the most affected biological processes in human enterocytes exposed to 3-NT+EA (9.15%, *p* < 0.01) (SG. 2C). Within this group, the most affected were error-free translation synthesis, error-prone translation synthesis and nucleotide-excision repair (DNA gap filling). The proliferating cell nuclear antigen (PCNA, fold change: 2.86), 70 kDa DNA-binding subunit of replication protein A (RPA1, fold change: 1.45), 14 kDa subunit of replication protein A (RPA3, fold change: 2.02), ribosomal protein S27a of ubiquitin-40S (RPS27A, fold change: 2.02), ribosomal protein L40 of ubiquitin-60S (UBA52, fold change: 2.02), polyubiquitin-B (UBB, fold change: 2.02), polyubiquitin-C (UBC, fold change: 2.02) and DNA ligase 3 (LIG3, only in 3-NT+EA) were the proteins found in higher quantity in this case (Table 2 and Tables S2–S9)

##### Oxidoreductase Activity

The oxidoreductase activity of enterocytes was also upregulated in 3-NT+EA-treated cells (7.84%, *p* < 0.01) (SG. 2C). Several relevant processes were affected, including dihydrotestosterone 17-beta-dehydrogenase activity, carnitine biosynthetic process, glyceraldehyde-3-phosphate dehydrogenase (NAD+) (non-phosphorylating) activity, aldehyde dehydrogenase NAD+, NAD(P)+ activity and the glycoside metabolic process, among others (Supplementary Table S9). The analysis of the molecular functions also revealed an up-regulation of the oxidoreductase activity acting on the CH-OH group of donors (19.35%, *p* < 0.01) (SG. 2D). In this case, it is worth emphasizing the up-regulation of the dihydrotestosterone 17-beta-dehydrogenase activity, glyceraldehyde-3-phosphate dehydrogenase (NAD+) (non-phosphorylating) activity, aldehyde dehydrogenase NAD+ and NAD(P)+ activity, and alcohol dehydrogenase (NADP+) activity (Supplementary Table S10). Some relevant enzymes accounting for these effects were member C3 of the aldo-keto reductase family 1 (AKR1C3, fold change: 3.21), type-2 3-hydroxyacyl-CoA dehydrogenase (HSD17B10, fold change: 1.42), mitochondrial delta-1-pyrroline-5-carboxylate dehydrogenase (ALDH4A1 fold change: 2.42), SHMT1, class-3 alcohol dehydrogenase (ADH5 1.71), fatty aldehyde dehydrogenase (ALDH3A2, fold change: 2.08), alcohol dehydrogenase 4 (ADH4, only in 3-NT+EA), alcohol dehydrogenase [NADP (+)] (AKR1A1, fold change: 1.24), aflatoxin B1 aldehyde reductase (member 2) (AKR7A2, fold change: 1.35), tissue alpha-L-fucosidase (FUCA1, fold change: 2.86) and alpha-N-acetylgalactosaminidase (NAGA, fold change: 1.64), among others (Table 2 and Tables S2–S9)

##### Aminopeptidase Activity

The aminopeptidase activity was one of the most affected molecular functions (11.29%, *p* < 0.01) (SG. 2D). The proteins found in higher abundance from this group were cathepsin B (CTSB, fold change: 1.72), aspartyl aminopeptidase (DNPEP, fold change: 1.98), dipeptidyl peptidase 2 (DPP7, fold change: 3.40), leukotriene A-4 hydrolase (LTA4H, fold change: 1.78), N-acetylated-alpha-linked acidic dipeptidase 2 (NAALAD2, fold change: 1.87) and lysosomal Pro-X carboxypeptidase (PRCP, fold change: 1.77) (Table 2 and Table S10).

##### Oxidoreductase Activity, Acting on a Sulfur Group of Donors

The analysis of the molecular functions also revealed up-regulation of oxidoreductases acting on the sulfur group of donors (6.45%, *p* < 0.01) (SG. 2D). Within this group, some relevant proteins which were significantly found in higher abundance were thioredoxin domain-containing protein 17 (TXNDC17, fold change: 2.19), thioredoxin (TXN, fold change: 1.73), mitochondrial glutaredoxin-related protein 5 (GLRX5, fold change: 1.33), mitochondrial glutathione reductase (GSR, fold change: 1.25), glutathione synthetase (GSS, fold change: 1.25), protein disulfide isomerase A4 (PDIA4, fold change: 1.21), selenium-binding protein 1 (SELENBP1, fold change: 1.75), and thioredoxin-like protein 1 (TXNL1, fold change: 1.44) (Table 2 and Tables S2–S10).

##### Other Proteins of Biological Significance Found in Higher Relative Quantity

Proteins of biological significance only found in 3-NT+EA treated cells that have not been included in the previous biological processes/molecular functions are alcohol dehydrogenase 4 (ADH4), glutathione S-transferase Mu 3 (GSTM3), ferroptosis suppressor protein 1 (AIFM2), heme oxygenase 1 (HMOX1) and subunit RPB1 of DNA-directed RNA polymerase II (POLR2A) (Table 2 and Table S2).

### 3.3. Endogenous Antioxidant Enzyme Activity

**Figure 3:**
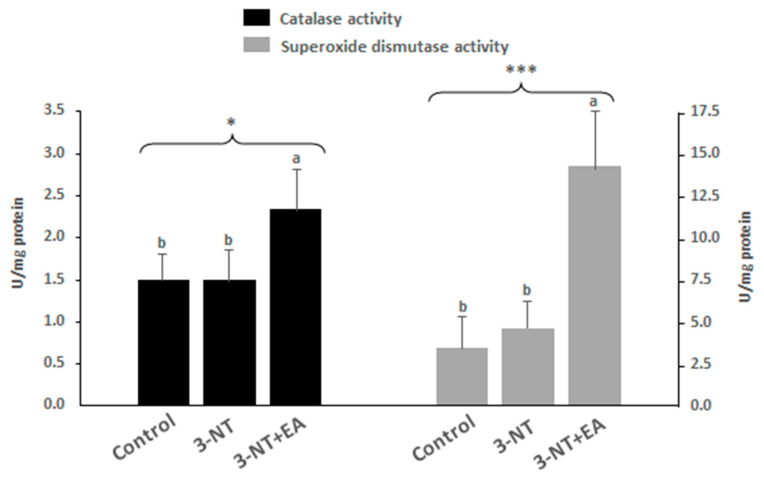
Antioxidant activity of catalase (black bars) and superoxide dismutase (grey bars) on differentiated human enterocytes upon exposure to 200 μM 3-NT and 200 μM 3-NT+200 μM EA for 72 h. Asterisks on top of bars denote significant differences between group of samples in ANOVA (* *p*<0.05; *** *p* < 0.001).(a–b) Different letters on top of bars denote significant differences between means in post-hoc Tukey tests (*p* < 0.05).

Further to the full proteome of the cells under study, the activity of two major antioxidant enzymes, namely, catalase (CAT) and superoxide dismutase (SOD) was analyzed to gain further insight into the physiological response of the cell to the exposure to the tested dietary species (Figure 3). Consistent with the proteome results, the catalase activity in cells exposed to 3-NT-EA was significantly higher than in those treated with 3-NT alone, or in control. While superoxide dismutase was not identified as discriminating between treatments, the assessment of its activity revealed that enterocytes challenged with 3-NT+EA had significantly higher SOD activity than the other two counterparts. 

## 4. Discussion

**Figure 4:**
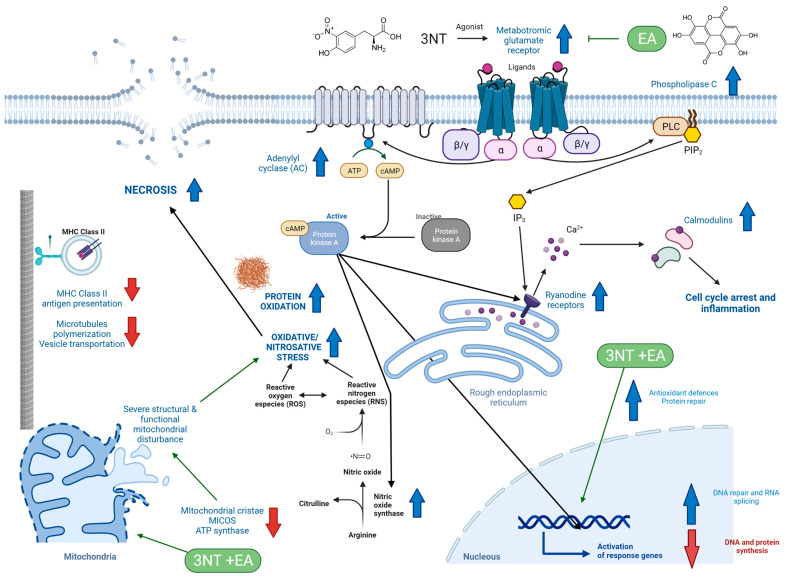
Proposal of underlying molecular mechanisms of the toxicological effects of 200 μM 3-NT and 200 μM 3-NT+200 μM EA for 72 h on differentiated human enterocytes. Upstream head blue arrows indicate higher concentration of proteins/upregulated biological processes in treated enterocytes. Downstream head red arrows indicate lower concentration of proteins/downregulated biological processes in treated enterocytes. Blunt head connectors indicate an inhibited biological process or metabolic pathway. Effect of EA is specifically denoted by green arrows. (For interpretation of the references to colour in this figure legend, the reader is referred to the web version of this article). The mechanisms and routes depicted in this Figure are proposed based on the interpretation of the data from cytometry, proteomics and accretion of protein oxidation (oxidative stress). Confirmation of the impairment of all these downstream paths and interconnections of complex cellular mechanisms requires further specific studies.

Presently, the onset of oxidative and nitrosative stress is associated with multiple pathologies [1,2]. Dietary oxidized amino acids have been documented to induce redox imbalance and impairment of organic functions in human cells [15,21]. According to our hypotheses, 3-NT could be implicated in the assorted health disorders attributed to the intake of processed cured meat products, while the application of recognized antioxidants such as EA could contribute to counteract the redox-related biological impairments of 3-NT. The results from the present study revealed that 3-NT is certainly implicated in the impairment of specific biological functions in human enterocytes; however, the exposure to EA was not found to counteract such impairments. Contrary to our initial hypothesis, the combination of 3-NT and EA led to a severe impairment of the proteome of the enterocytes with remarkable increase in necrosis. Such cell injure can be visualized in the Picture S1 (Available as Supplementary Material). The most relevant biological impairments caused by 3-NT and 3-NT+EA in differentiated human enterocytes are discussed as follows. Figure 4 illustrates the metabolic pathways affected in these cells by the exposure to 3-NT and 3-NT+EA. 

### 4.1. Impact of 3-NT and 3-NT+EA on Calmodulin-Dependent Intracellular Signaling Pathway 

The calmodulin protein family is a group of biologically relevant proteins, which play a central role in Ca^2+^ cell sensing, ion channel regulation and molecular signaling pathways with multiple physiological implications [22]. The higher relative quantity of 3 calmodulin proteins suggests that 3-NT exposure would have, as a primary molecular mechanism, the impairment of downstream biological processes depending on such proteins. As identified by CLUEGO, 3-NT upregulated the cyclic nucleotide phosphodiesterase activity and type 3 metabotropic glutamate receptor binding activity, which directly affects the nitric oxide synthase regulator activity, the adenylate cyclase activator activity, the regulation of ryanodine-sensitive calcium-release channel activity and the N-terminal myristoylation domain binding. These processes and functions accounted for around 87% of all proteomic upregulations caused by 3-NT exposure, which highlights the relevance of these molecular mechanisms in the potential toxicological effects of this nitrosated species. 

Type 3 metabotropic glutamate receptors are constituted by seven transmembrane domains coupled to the G-protein signaling system [23]. The adenylate cyclase activator activity enables the formation of cAMP from ATP [24]. On the other hand, cyclic nucleotide phosphodiesterase activity is responsible for eliminating the excess of cAMP in the cell by converting it into 5′-AMP [24]. cAMP activates the catalytic subunits of protein kinase A (PKA), which goes to the nucleus, activating transcription factors [25]. PKA is also involved in the activation of ryanodine receptors (RYRs) [26], which can also be activated by IP3 through the phospholipase C pathway [27]. RYRs release calcium from rough endoplasmic reticulum (RER) to the cytosol, which, in turn, activates calmodulin proteins [26]. All these processes seemed to be increased by the effect of 3-NT exposure, and the consequences of such up-regulation have been described as harmful for enterocytes. In a recent study, Cunningham et al. [28] reported that activation of the calmodulin downstream molecular pathway in intestinal cells is initiated by an epithelial injury and leads to cell cycle arrest and inflammation processes. The inactivation of such molecular pathway protected against murine inflammatory bowel disease and colitis. It is, therefore, reasonable to consider that the higher abundance of calmodulin proteins found in enterocytes when treated with 3-NT may have harmful biological consequences. Consistently, proteins corresponding to DNA repair and RNA splicing were in higher quantities as compared to control counterparts. 3-NT-treated enterocytes showed impaired processes responsible for carrying out a normal synthesis of DNA and proteins (Table 1). These results are compatible with impaired cell cycle regulation. Consistently with our current study, Santulli et al. [27] observed the activation of RyRs by oxidative and nitrosative species and emphasized that these molecular mechanisms are implicated in the onset of various pathological conditions such as diabetes mellitus, hypertension and skeletal muscle disorders, among others.

3-NT is also involved in increasing nitric oxide synthase regulatory activity. Nitric oxide synthase (NOS) is an enzyme that catalyzes the conversion of L-arginine to L-citrulline producing nitric oxide (NO) [29], and has a binding domain to calmodulin. The cyclic-nucleotide phosphodiesterase activity, also increased in 3-NT-treated cells, is involved in the activation of inducible nitric oxide synthase (iNOS) via adenylate cyclase/protein kinase A dependent of stimulatory G-protein [29]. At high levels of NO, or in the presence of ROS, the formation of peroxynitrite and its potential cytotoxic effects are enhanced [30]. Peroxynitrite can damage a variety of molecules in cells, including DNA and proteins, leading to apoptosis and necrosis [30], a situation which, in fact, occurred in the present study. Peroxynitrite plays a crucial role in chronic inflammatory diseases, diabetes, cancer and neurodegenerative disorders, among others [30], affect T cells and negatively influence the immune response [31], corresponding with the aforementioned obtained results, where MHC class II-restricted antigen presentation for CD4^+^ T cell-dependent was affected. Currently, 3-NT is recognized as a biomarker of up-regulated inducible NO synthase in inflammation [32]. Interestingly, 3-NT, a product of the nitrosative stress to proteins, would contribute to inducing further nitrosative stress by the activation of specific metabolic routes. 

The molecular function of N-terminal myristoylation domain binding is affected by 3-NT exposure. N-myristylation of proteins is a co-translational lipidic modification of many eukaryotic proteins [33]. Myristoylation directly regulates the biological activity of endothelial nitric oxide synthase (eNOS), increasing calcium flows, and activating the calcium/calmodulin complex (calcium/CaM). This process is involved in the regulation of cell-signaling pathways in biological processes such as carcinogenesis and immune function [33]. Thus, for all of the above mentioned, 3-NT can be considered as an agonist of type 3 metabotropic glutamate receptors in cells, which stimulates different relevant pathways. Acting as such, 3-NT promotes the deregulation of cAMP and calcium metabolism, which affects, in turn, the normal operation of the immune system through calmodulin. 

The results obtained in EA-treated cells were coherent with those found in the literature, in which the ability of EA to enhance calcium metabolism, apoptosis and necrosis in tumor cells, is reported (revised by Mohammadinejad et al., [34]). However, enterocytes treated with EA led to an attempt to reverse some of the above-mentioned impaired biological functions (Supplementary Table S8). It was ignored whether a higher dose of EA would be necessary to compensate the effects of 3-NT on these pathways. Yet, the injurious effects of the combination of both species at these concentrations on enterocytes in terms of oxidative stress and necrosis, discussed in due course, suggest that, unlike our initial hypothesis, EA does not exert a protective effect against 3-NT-induced toxicity, but the opposite.

### 4.2. Impact of 3-NT and 3-NT+EA on Immune System and Steroid Hormones

The immune system is a network of cells and biological processes that protects an organism from internal and external hazardous substances. The major histocompatibility complex (MHC) is involved in the codification of proteins found on the surface of the cell recognizing potential antigens. Antigenic peptide-loaded MHC class II molecules (peptide–MHC class II) are expressed on the surface of professional antigen-presenting cells (APCs) such as macrophages, B cells, dendritic cells and thymic epithelial cells [35]. As the primary barrier between human organism and its environment, intestinal epithelial cells, as observed in the present study, express MHC class II in their surface, although the functional consequences of this expression are not fully understood [36]. All these cells present the antigen to antigen-specific CD4^+^ T cells, a mechanism that is essential for a specific and effective immune response [35]. In this study, processes involved in the antigen processing and presentation of exogenous peptide antigen via MHC class II were affected by the exposure of 3-NT. Proteins implicated in the motility of vesicles through microtubules (MYH10) [37], blebs formation (ROCK2) [38], vesicle trafficking (SNX9) [39], reorganization of actin cytoskeleton, exocytosis and early steps of protein synthesis (EIF2S3, EIF2S3B and EIF3D) [40] (Table 1) were significantly reduced in abundance in the presence of 3-NT compared to control counterparts. The lower relative abundance of tau protein binding function in 3-NT-treated cells is consistent with the impairment of the MHC class II mediated process (Supplementary Table S4). The tau protein binding was decreased in DCTN1 protein, which is an essential cofactor involved in most cellular functions of the microtubule motor cytoplasmic dynein [41] and in GSK3B protein, an essential key in protein tau phosphorylation. It is, hence, reasonable to hypothesize that the decrease of microtubule polymerization prevents vesicles of antigenic peptide-loaded MHC class II molecules from reaching the cell surface. Other proteins related to immunity, such as IRF3 [42] or LRBA [43], were also found in lower quantities in the presence of dietary concentrations of 3-NT. These results suggest that 3-NT impairs the ability of enterocytes to act as APCs and effectively contribute to protection against biological hazards. This could be explained by i) an impaired synthesis of proteins related to immunity, and ii) an impaired transportation of such proteins to the cell surface. These results are consistent with those published by Birnboim et al. [32] and Ahsan [44], where 3-NT is linked to systemic autoimmunogenic conditions and is related to diseases associated with immunological reactions where formation of Tyr-nitrated proteins has a major role [45]. Thus, 3-NT has a toxicological effect in the immune system through its implication in the decrease of MHC class II-restricted antigen presentation for CD4+ T cell-dependent, which is, as aforementioned, necessary for a suitable immune response. In addition, the results obtained revealed that the incorporation of EA to enterocytes did not seem to have an impact on this biological impairment caused by 3-NT. 

In relation to steroid hormones, the proteins HSD3B1 and HSD3B2, involved in oxidation and isomerization in the biosynthesis of hormonal steroids [46], were not found in 3-NT treated cells. Steroid hormones are involved in a number of processes such as immune functions, inflammation, the control of metabolism and sexual development through sex steroids and corticosteroids [47]. Interestingly, protein HSD17B10 was found in terms dihydrotestosterone 17-beta-dehydrogenase activity and lipid oxidation (Supplementary Table S9) in higher abundance in 3-NT+EA-treated cells. This protein, with hydroxysteroid dehydrogenase activity in steroid hormones [48], may be positive to cells as it may counteract the drop in proteins HSD3B1 and HSD3B2 caused by 3-NT. Therefore, EA may enable physiological biosynthesis of hormonal steroids in enterocytes and counteract, at some extent, the impairment caused by 3-NT. 

### 4.3. Impact of 3-NT and 3-NT+EA on Antioxidant Defenses and Oxidative Stress 

The analysis of specific protein oxidation markers in enterocytes revealed that the combination of 3-NT and EA remarkably promoted the onset of oxidative stress in enterocytes. These indicators of the oxidative damage to proteins are formed as a result of the oxidative deamination of alkaline amino acids (i.e., lysine) in the presence of ROS [49]. It is known that decreased and inefficient mitochondrial activity is linked to ROS generation, and, consequently, to oxidative stress [50]. Enterocytes exposed to 3-NT+EA showed a significant decrease in mitochondrial cristae formation and a lower abundance in proteins related to mitochondrial ATP synthase. The proteins found in lower abundance in 3-NT+EA-treated cells were related to the MICOS complex (mitochondrial contact site and cristae organizing system) (Table 2). MICOS is a multi-subunit complex found in the inner mitochondrial membrane. Mitochondrial function and its architecture are closely related because an anomalous mitochondrial architecture leads to mitochondrial dysfunction [51]. MICOS is involved in human diseases such as amyotrophic lateral sclerosis, Alzheimer’s and Parkinson’s disease [51]. In agreement with the present results, several previous studies reported that EA inhibits mitochondrial respiration and had a negative impact on the restoration of both GSH and ATP, and on mitochondrial complex II function [52,53]. The remarkable impact of EA in promoting oxidative stress in 3-NT-treated enterocytes (as measured by protein oxidation) could be a plausible consequence of the disturbance effect of the phytochemical on mitochondrial activity. This could be one likely mechanism behind the profusely reported pro-oxidant activity of ellagic acid [54]. It is known that such pro-oxidant action depends on a number of factors, including the dose and occurrence of oxygen molecules and transition metals [54]. The present study reveals that 3-NT activates this pro-oxidant mechanism as exposure to EA alone, had, in general, protective effects on enterocytes against oxidative stress, apoptosis and necrosis (Figure 1E–G). The molecular mechanism by which the combination of both species leads to such severe oxidative stress and necrosis is yet to be elucidated. 

The oxidative damage to proteins measured in 3-NT+EA cells apparently contrasts with the lack of differences in ROS as assessed by flow cytometry. The lack of correspondence between both measurements is consistent with a previous study performed in human enterocytes exposed to 3-NT [9]. It is reasonable to hypothesize that, at the point of cell harvesting, ROS were already depleted while the effects of their pro-oxidative effects (oxidative damage and necrosis) were noticeable. Furthermore, the probe employed for cytometric ROS measurement detects, specifically, superoxide radicals, concentration of which commonly reflects mitochondrial activity. An impaired mitochondrial respiration is, in fact, compatible with the present results, which would actually explain the creation of a cellular pro-oxidative environment with other ROS that would, in turn, lead to the remarkable accretion of oxidized proteins. 

The effect of 3-NT+EA on promoting the occurrence of detoxifying enzymes such as GSS and GSR (both involved in glutathione metabolism), GSTM3 and CAT in enterocytes, supports the hypothesis of enterocytes reinforcing the endogenous antioxidant defenses in response to the pro-oxidative environment caused by the combination of both chemical species. These enzymes are known to increase in cells in response to oxidative stress [55,56]. Furthermore, ADH4, which is well known for playing a relevant role in detoxifying ROS in enterocytes as a primary barrier to dietary pro-oxidative species [57], was only found in cells exposed to both 3-NT and EA. The proteomic results are consistent with additional analysis of the endogenous antioxidant activity of CAT and SOD. The activity of both enzymes was significantly higher in cells treated with 3-NT`+EA than in the other two groups of cells (3-NT, control). However, this attempt to strengthen the endogenous antioxidant defenses failed, as observed in the extent of oxidative damage to proteins and the severe necrosis found in enterocytes exposed to 3-NT+EA.

### 4.4. Impact of 3-NT and 3-NT+EA in Cell Viability, Apoptosis, Necrosis and Protein Repair 

The flow cytometry assessment revealed that 3-NT caused a significant decrease in cell viability (*p* < 0.05) and a significant increase in necrosis (*p* < 0.05) (Figure 1A). As discussed above, the cytotoxicity exerted by 3-NT on enterocytes may be derived from the up-regulation of calcium-dependent biological functions that may have caused physiological impairments, including the onset of oxidative stress. While the increase of ROS was not detected by flow cytometry in 3-NT-treated cells, the accretion of oxidized proteins was significant as compared to CONTROL cells. The up-regulation of type 3 metabotropic glutamate receptors in the presence of 3-NT led to a higher abundance in a number of enzymes involved in important pathways of cellular metabolism, including nitric oxide synthase. This enzyme increases the amount of NO in cells, which makes the formation of RNS species possible, along with 3-NT, such as peroxynitrite. This species can damage DNA and proteins, increasing apoptosis and necrosis [30,31], with necrosis being the most characteristic mechanism induced by peroxinitrite [30]. In a previous study [9], a significant decrease of cell viability was accompanied by a significant increase in necrosis. Other studies have reported the same situation in cell cultures and animal models [8,58]. 

The significant decrease in cell viability (*p* < 0.05) in enterocytes exposed to 3-NT and EA, is reflected in a remarkable significant increase in necrosis compared to 3-NT treated cells (*p* < 0.01) (Figure 1C). Cells exposed to 3-NT and EA seemed to enter into necrosis, which shows the severe chemical insult caused by the combination of these two chemical species. On the other hand, the significant decrease in apoptosis (*p* < 0.01) may be caused by of the ability of EA to counteract the upregulation of type 3 metabotropic glutamate receptors caused by 3-NT. Yet, the most plausible means of cytotoxicity caused by 3-NT+EA, which would explain the remarkable increase in oxidized proteins and cell necrosis, would be the severe mitochondrial disturbance. Such physiological impairment was manifested in the proteome by downregulation of the MICOS complex and other proteins implicated in the mitochondrial respiratory chain (Table 2). As aforementioned, an abnormal mitochondrial conformation leads to mitochondrial dysfunction [51] and the onset of oxidative stress, which would explain, in turn, the significant and remarkable accretion of oxidized proteins and increased necrosis. Indeed, the protein CHCHD3, which plays an important role in maintaining the stability of the MICOS complex and the morphology of mitochondrial cristae [59], is remarkably diminished in 3-NT+EA-treated enterocytes along with other proteins related to this complex (Table 2). 

Finally, the process of protein repair was also affected by the exposure to 3-NT+EA. Proteins related to the proteasome complex involved in the proteolytic degradation of most intracellular proteins [60] were found in higher abundance in the presence of 3-NT+EA-treated cells. Other proteins involved in post-replication repair and DNA damage checkpoint activation, such as RPA3 [61], were also increased in quantity as compared to 3-NT-treated cells. These results are coherent with the aforementioned attempt of the enterocytes to counteract the severe oxidative insult induced by the combination of 3-NT and EA. These efforts were, however, insufficient to avoid massive cell necrosis caused by these two chemical species. 

## 5. Conclusions

We describe, for the first time, the underlying molecular mechanisms of the toxicological effects of 3-NT in human enterocytes. While this proteomics-based approach enables the study of these noxious effects from a comprehensive perspective, the confirmation of the impairment of all these downstream paths and interconnections of complex cellular mechanisms requires further specific studies. According to the generated data, the nitrosated amino acid 3-NT impairs several calcium-dependent physiological processes and affects the ability of enterocytes to act as antigen-presenting cells, compromising the immune response against potential biological threats. While it could be thought that EA would revert some of these mechanisms, the combination of 3-NT and the phytochemical led to compromised cell viability through anomalous mitochondria conformation and functionality, leading to severe oxidative stress and massive cell necrosis. These results highlight the importance of the study of dietary nitrosated amino acids in intestinal cell physiology, as they commonly occur in processed meat/dairy products where nitrite is used. Furthermore, the toxicological effects of 3-NT+EA should be studied for potential therapeutic studies in which cell death is deliberately induced. 

## Figures and Tables

**Table 1:** Proteins from differentiated human enterocytes affected by the exposure to 200 μM 3-nitrotyrosine for 72 h in comparison to the non-treated control (C).

Protein Name	Gene Name	*p*-Value	Fold-Change ^1^	Biological Function	FASTA Accession Number
Rho-associated protein kinase 2	*ROCK2*	-	C	Involved in blebs formation	O75116
STAM-binding protein	*STAMBP*	-	C	Associated with intracellular accumulation of ubiquitinated protein	O95630
3 beta-hydroxysteroid dehydrogenase/Delta 5-->4-isomerase type 1	*HSD3B1*	-	C	Oxidation and isomerization in the biosynthesis of hormonal steroids	P14060
3 beta-hydroxysteroid dehydrogenase/Delta 5-->4-isomerase type 2	*HSD3B2*	-	C	Oxidation and isomerization in the biosynthesis of hormonal steroids	P26439
Glycogen synthase kinase-3 beta	*GSK3B*	-	C	Protein tau phosphorylation	P49841
Arf-GAP with Rho-GAP domain, ANK repeat and PH domain-containing protein 1	*ARAP1*	-	C	Reorganization of actin cytoskeleton	Q96P48
DNA-directed RNA polymerase II subunit RPB1	*POLR2A*	-	C	Transcription of DNA into RNA	P24928
Exocyst complex component 5	*EXOC5*	-	C	Involved in exocytosis	O00471
Alcohol dehydrogenase 4	*ADH4*	-	C	Oxidoreductase activity	A0A0D9SFB5
Developmentally regulated GTP-binding protein 2	*DRG2*	-	C	Maintenance of cell structure and protein trafficking	P55039
STAM-binding protein	*STAMBP*	-	C	Signal transduction for cell growth	O95630
Cirhin	*CIRH1A*	-	C	Pre-ribosomal RNA transcription	L0R599
F-actin-capping protein subunit beta	*CAPZB*	-	C	Cytoskeletal organization	P47756
Coronin-2A	*CORO2A*	-	C	Actin filament binding	Q92828
U6 snRNA-associated Sm-like protein LSm2	*LSM2*	-	C	Involved in RNA-binding proteins	Q9Y333
Cytoplasmic dynein 1 heavy chain 1	*DYNC1H1*	0.044	0.53	Motility of vesicles through microtubules	Q14204
Delta (24)-sterol reductase	*DHCR24*	0.037	0.55	Protection against oxidative stress and apoptosis	Q15392
Interferon regulatory factor 3	*IRF3*	0.020	0.63	Related to immunity	Q14653
Myosin-10	*MYH14*	0.036	0.68	Cytoskeleton reorganization	P35580
Lipopolysaccharide-responsive and beige-like anchor protein	*LRBA*	0.026	0.70	Vesicle trafficking of immune effector	P50851
AP-1 complex subunit sigma-1A	*AP1S1*	0.002	0.71	Related to clathrin	P61966
DNA-directed RNA polymerase II subunit RPB2	*POLR2B*	0.008	0.71	Component of RNA polymerase II	P30876
Histone H2B tipo 1-L	*H2BC13*	0.048	0.73	Histone related to nucleosome	Q99880
Histone H2B type 1-M	*H2BC14*	0.048	0.73	Histone related to nucleosome	Q99879
Histone H2B type 1-N	*H2BC15*	0.048	0.73	Histone related to nucleosome	Q99877
Histone H2B type 2-F	*H2BC18*	0.048	0.73	Histone related to nucleosome	Q5QNW6
Histone H2B type 1-D	*H2BC5*	0.048	0.73	Histone related to nucleosome	P58876
Histone H2B type 1-H	*H2BC9*	0.048	0.73	Histone related to nucleosome	Q93079
D-dopachrome decarboxylase	*DDT*	0.024	0.74	Tautomerization of D-dopachrome	P30046
D-dopachrome decarboxylase-like protein	*DDTL*	0.024	0.74	Lyase activity	A6NHG4
Eukaryotic translation initiation factor 3 subunit D	*EIF3D*	0.022	0.75	Early steps of protein synthesis	O15371
Sorting nexin-9	*SNX9*	0.009	0.75	Intracellular vesicle trafficking	O75116
SWI/SNF-related matrix-associated actin-dependent regulator of chromatin subfamily A member 5	*SMARCA5*	0.049	0.78	Helicase involved in nucleosome-remodeling activity	O60264
Ribosome-binding protein 1	*RRBP1*	0.017	0.78	Mediates interaction between ribosome and endoplasmic reticulum membrane	Q9P2E9
Coatomer subunit alpha	*COPA*	0.026	0.79	Formation of blebs in Golgi apparatus	P53621
Dynactin subunit 1	*DCTN1*	0.046	0.82	Cofactor involved in microtubule motor cytoplasmic dynein	Q14203
Eukaryotic translation initiation factor 2 subunit 3	*EIF2S3*	0.045	0.86	Early steps of protein synthesis	P41091
Eukaryotic translation initiation factor 2 subunit 3B	*EIF2S3B*	0.045	0.86	Early steps of protein synthesis	Q2VIR3
Beta-centractin	*ACTR1B*	0.026	0.87	Motility of vesicles through microtubules	P42025
Leukocyte elastase inhibitor	*SERPINB1*	0.037	0.87	Inflammatory caspase activation	P30740
Thioredoxin reductase 1, cytoplasmic	*TXNRD1*	0.032	1.15	Regulation of cellular redox and reductase activity on H_2_O_2_	Q16881
Calmodulin-1; Calmodulin-2; Calmodulin-3	*CALM1, CALM2, CALM3*	0.009	1.29	Involved in nitric oxide synthase regulator activity, adenylate cyclase activator activity and positive, negative regulation of ryanodine-sensitive calcium-release channel activity, type 3 metabotropic glutamate receptor binding and N-terminal myristoylation domain binding	P0DP23; P0DP24; P0DP25
Mitochondrial glutamate carrier 1	*SLC25A22*	0.002	1.30	Mitochondrial carrier of glutamate	E9PJH7
60S ribosomal protein L11	*RPL11*	0.033	1.57	Component of the ribosome	P62913
Cadherin-17	*CDH17*	0.009	1.71	Calcium-dependent cell adhesion protein involved in intestinal peptide transport	Q12864
Tumor susceptibility gene 101 protein	*TSG101*	0.045	2.00	Regulator of vesicular trafficking process	Q99816
Serine/arginine repetitive matrix protein 1	*SRRM1*	0.042	2.13	Involved in numerous pre-mRNA processing events, part of pre- and post-splicing	Q8IYB3
Neutral amino acid transporter B (0)	*SLC1A5*	0.005	2.22	Sodium-dependent amino acids transporter including glutamine	Q15758
U1 small nuclear ribonucleoprotein C	*SNRPC*	-	3-NT	Component of the spliceosome	P09234
CD2 antigen cytoplasmic tail-binding protein 2	*CD2BP2*	-	3-NT	Involved in pre-mRNA splicing	O95400
Carcinoembryonic antigen-related cell adhesion molecule 1	*CEACAM1*	-	3-NT	Coinhibitory receptor in immune response	P13688
La-related protein 4B	*LARP4B*	-	3-NT	mRNA translation	Q92615
Lipid droplet regulating VLDL assembly factor AUP1	*AUP1*	-	3-NT	Translocation of misfolded proteins and degradation by the proteasome	Q9Y679
Non-homologous end-joining factor 1	*NHEJ1*	-	3-NT	DNA repair	Q9H9Q4
DNA-directed RNA polymerase II subunit RPB3	*POLR2C*	-	3-NT	Transcription of DNA into RNA	P19387

^1^ Fold change (FC) indicates the degree of quantity change for a particular protein between cells (control vs. 3-NT); FC <1 denotes a decrease in the concentration of protein in the treated sample (vs. control); FC >1 indicates a significant increase in the concentration of the protein in treated sample (vs. control). When a given protein is only present in one of the groups, fold change cannot be measured and such condition is denoted as “C” (if protein is only present in C cells) or 3-NT (if protein is only present in 3-NT-exposed cells). 3-NT3-NT.

**Table 2:** Proteins from differentiated human enterocytes affected by the exposure to 200 μM 3-nitrotyrosine + 200 μM ellagic acid for 72 h in comparison to those exposed to 200 μM 3-nitrotyrosine for 72 h.

Protein Name	Gene Name	p-Value	Fold Change ^1^	Biological Function	FASTA Accession Number
Translation initiation factor eIF-2B subunit alpha	*EIF2B1*	-	3-NT	Catalyzes the exchange of eukaryotic initiation factor 2-bound GDP for GTP	Q14232
Eukaryotic translation initiation factor 2 subunit 2	*EIF2S2*	-	3-NT	Involved in the early steps of protein synthesis along with GTP and tRNA	P20042
La-related protein 1	*LARP1*	-	3-NT	RNA-binding protein	Q6PKG0
ATP synthase subunit delta, mitochondrial	*ATP5F1D*	-	3-NT	Component of mitochondrial membrane ATP synthase	P30049
MICOS complex subunit MIC13	*MICOS13*	-	3-NT	Component of the MICOS complex, formation and maintenance of mitochondrial cristae	Q5XKP0
Mitochondrial inner membrane protein OXA1L	*OXA1L*	-	3-NT	Insertion of integral membrane proteins into the mitochondrial inner membrane	Q15070
ATP-binding cassette sub-family B member 10, mitochondrial	*ABCB10*	-	3-NT	Exporting from the mitochondrial matrix to the cytosol in an ATP-dependent manner	Q9NRK6
Ribosome-releasing factor 2, mitochondrial	*GFM2*	-	3-NT	Mitochondrial GTPase involved in mitochondrial protein biosynthesis	Q969S9
Phosphatidylinositol-binding clathrin assembly protein	*PICALM*	-	3-NT	Cytoplasmic adapter protein related to clathrin-mediated endocytosis	Q13492
Plasma membrane calcium-transporting ATPase 4	*ATP2B4*	-	3-NT	Calcium/calmodulin-regulated enzyme, catalyzes hydrolysis of ATP coupled with transport of calcium out of the cell	P23634
Epidermal growth factor receptor	*EGFR*	-	3-NT	Receptor tyrosine kinase binding	P00533
MICOS complex subunit MIC19	*CHCHD3*	1.41 × 10^−5^	0.07	Component of the MICOS complex, maintenance of mitochondrial crista junctions	Q9NX63
Histone H1.4	*H1-4*	1.00 × 10^−4^	0.08	Nucleosome assembly	P10412
ATP synthase subunit alpha, mitochondrial	*ATP5F1A*	0.010	0.17	Component of mitochondrial membrane ATP synthase	P25705
60S ribosomal protein L13	*RPL13*	0.011	0.22	Component of the ribosome	P26373
Dynamin-2	*DNM2*	0.014	0.26	Microtubule-associated protein, able to bind and hydrolyze GTP	P50570
60S ribosomal protein L23a	*RPL23A*	1.80 × 10^−3^	0.26	Component of the ribosome	P62750
60S ribosomal protein L12	*RPL12*	1.86 × 10^−6^	0.27	Component of the ribosome	P30050
ATP synthase subunit beta, mitochondrial	*ATP5F1B*	0.026	0.31	Component of mitochondrial membrane ATP synthase	P06576
40S ribosomal protein S7	*RPS7*	0.003	0.33	Component of the ribosome	P62081
DnaJ homolog subfamily C member 11	*DNAJC11*	0.004	0.33	Component of the MICOS complex, mitochondrial inner membrane organization	Q9NVH1
Dynamin-like 120 kDa protein, mitochondrial	*OPA1*	4.21 × 10^−5^	0.34	Regulates the equilibrium between mitochondrial fusion and mitochondrial fission	O60313
Stomatin-like protein 2, mitochondrial	*STOML2*	2.00 × 10^−4^	0.38	Mitochondrial protein involved in the activity of mitochondria	Q9UJZ1
Adenylate kinase 4, mitochondrial	*AK4*	3.07 × 10^−6^	0.38	Interconversion of nucleoside phosphates	P27144
EF-hand domain-containing protein 1, mitochondrial	*LETM1*	1.60 × 10^−4^	0.40	Mitochondrial proton/calcium antiporter, assembly of the supercomplexes of the respiratory chain	O95202
Monocarboxylate transporter 1	*SLC16A1*	9.00 × 10^−4^	0.41	Proton-coupled monocarboxylate transporter	P53985
ATP synthase subunit O, mitochondrial	*ATP5PO*	2.39 × 10^−5^	0.42	Component of mitochondrial membrane ATP synthase	P48047
Eukaryotic initiation factor 4A-II	*EIF4A2*	9.13 × 10^−5^	0.43	Required for mRNA binding to ribosome	Q14240
Eukaryotic translation initiation factor 4E	*EIF4E*	0.026	0.46	Involved in protein synthesis and ribosome binding	P06730
ATP synthase subunit d, mitochondrial	*ATP5PD*	5.35 × 10^−5^	0.46	Component of mitochondrial membrane ATP synthase	O75947
MICOS complex subunit MIC60	*IMMT*	4.74 × 10^−6^	0.47	Component of the MICOS complex, maintenance of mitochondrial cristae morphology	Q16891
60S ribosomal protein L11	*RPL11*	0.035	0.49	Component of the ribosome	P62913
ATP synthase subunit gamma, mitochondrial	*ATP5F1C*	3.1 × 10^−4^	0.50	Component of mitochondrial membrane ATP synthase	P36542
ATP synthase F (0) complex subunit B1, mitochondrial	*ATP5PB*	1.63 × 10^−5^	0.50	Component of mitochondrial membrane ATP synthase	P24539
AFG3-like protein 2	*AFG3L2*	4.1 × 10^−3^	0.50	Involved in the regulation of OPA1	Q9Y4W6
Sorting and assembly machinery component 50 homolog	*SAMM50*	0.003	0.60	Maintenance of the structure of mitochondrial cristae and the assembly of the mitochondrial respiratory chain complexes	Q9Y512
Cytochrome c oxidase subunit 5B, mitochondrial	*COX5B*	1.9 × 10^−3^	0.61	Component of the cytochrome c oxidase	P10606
Calcium/calmodulin-dependent protein kinase type II subunit beta	*CAMK2B*	0.014	0.66	Calcium/calmodulin-dependent protein kinase	Q13554
Guanine nucleotide-binding protein G(s) subunit alpha isoforms XLas	GNAS	0.008	0.67	Transducer in pathways controlled by G protein-coupled receptors, activation of adenylate cyclase	Q5JWF2
Heat shock protein HSP 90-beta	*HSP90AB1*	3.13 × 10^−6^	0.68	Chaperone involved in cell cycle control and signal transduction	P08238
Pyruvate kinase PKM	*PKM*	4.1 × 10^−4^	0.7	Glycolytic enzyme generating ATP and translation regulator of endoplasmic reticulum-associated ribosomes	P14618
RNA-binding protein 4	*RBM4*	0.007	0.73	Splicing of pre-mRNA and translation regulation	Q9BWF3
Calmodulin-1; Calmodulin-2; Calmodulin-3	*CALM1; CALM2; CALM3*	0.022	0.73	Nucleotide binding, 3 metabotropic glutamate receptor binding, N-terminal myristoylation domain binding, adenylate cyclase activator activity and nitric oxide synthase regulator activity	P0DP23; P0DP24; P0DP25
Calcium-binding mitochondrial carrier protein Aralar2	*SLC25A13*	4.1 × 10^−4^	0.74	Mitochondrial and calcium-binding carrier, exchange cytoplasmic glutamate with mitochondrial aspartate	Q9UJS0
Heat shock protein HSP 90-alpha	*HSP90AA1*	0.008	0.82	Chaperone involved in cell cycle control and signal transduction	P07900
Proteasome subunit alpha type 1	*PSMA1*	0.009	1.18	Component of proteasome complex involved in the proteolytic degradation of most intracellular proteins	P25786
Proteasome subunit beta type-5	*PSMB5*	0.002	1.19	Component of proteasome complex involved in the proteolytic degradation of most intracellular proteins	P28074
Protein disulfide-isomerase A4	*PDIA4*	0.017	1.21	Catalyzes the rearrangement of -S-S- bonds	P13667
Aldo-keto reductase family 1 member A1	*AKR1A1*	0.013	1.24	Catalyzes the NADPH-dependent reduction of carbonyl-containing compounds, detoxifying enzyme	P14550
Glutathione synthetase	*GSS*	0.039	1.25	Catalyzes the production of glutathione	P48637
Glutathione reductase, mitochondrial	*GSR*	0.007	1.25	Maintains high levels of reduced glutathione in the cytosol	P00390
Proteasome subunit beta type 4	*PSMB4*	0.015	1.32	Non-catalytic component of proteasome complex involved in the proteolytic degradation of most intracellular proteins	P28070
Glutaredoxin-related protein 5, mitochondrial	*GLRX5*	0.032	1.33	Involved in mitochondrial iron-sulfur cluster transfer	Q86SX6
Serine hydroxymethyltransferase, mitochondrial	*SHMT2*	0.003	1.34	Catalyzes the cleavage of serine to glycine	P34897
Aflatoxin B1 aldehyde reductase member 2	*AKR7A2*	0.002	1.35	NADPH-dependent aldehyde reductase activity	O43488
Proteasome subunit beta type 1	*PSMB1*	0.002	1.40	Non-catalytic component of proteasome complex involved in the proteolytic degradation of most intracellular proteins	P20618
3-hydroxyacyl-CoA dehydrogenase type-2	*HSD17B10*	0.007	1.42	Hydroxysteroid dehydrogenase activity in steroid hormones	Q99714
Thioredoxin-like protein 1	*TXNL1*	1.5 × 10^−4^	1.44	Disulfide oxidoreductase activity	O43396
Replication protein A 70 kDa DNA-binding subunit	*RPA1*	1.4 × 10^−4^	1.45	DNA replication and response to DNA damage	P27694
Sorbitol dehydrogenase	*SORD*	1.3 × 10^−3^	1.46	Catalyzes the reversible NAD^+^-dependent oxidation of sugar alcohols, a key enzyme in the polyol pathway	Q00796
Proteasome subunit beta type 3	*PSMB3*	0.008	1.55	Non-catalytic component of proteasome complex involved in the proteolytic degradation of most intracellular proteins	P49720
Proteasome subunit alpha type 6	*PSMA6*	9.00 × 10^−4^	1.62	Component of proteasome complex involved in the proteolytic degradation of most intracellular proteins	P60900
Alpha-N-acetylgalactosaminidase	*NAGA*	0.004	1.64	Involved in the breakdown of glycolipids	P17050
Alcohol dehydrogenase class-3	*ADH5*	0.021	1.71	Catalyzes the oxidation of S-(hydroxymethyl) glutathione	P11766
Cathepsin B	*CTSB*	0.016	1.72	Catabolism of intracellular proteins	P07858
Thioredoxin	*TXN*	0.001	1.73	Involved in antioxidant reactions	P10599
Serine hydroxymethyltransferase, cytosolic	*SHMT1*	2.3 × 10^−4^	1.74	Interconversion of serine and glycine	P34896
Selenium-binding protein 1	*SELENBP1*	0.006	1.75	Catalyzes the oxidation of methanethiol	Q13228
Lysosomal Pro-X carboxypeptidase	*PRCP*	0.005	1.77	Cleavage of amino acids	P42785
Leukotriene A-4 hydrolase	*LTA4H*	6.06 × 10^−5^	1.78	Aminopeptidase activity	P09960
Proteasome subunit beta type 2	*PSMB2*	0.008	1.79	Non-catalytic component of proteasome complex involved in the proteolytic degradation of most intracellular proteins	P49721
Succinate dehydrogenase [ubiquinone] iron–sulfur subunit, mitochondrial	*SDHB*	1.7 × 10^−3^	1.87	Iron–sulfur protein subunit of succinate dehydrogenase, involved in complex II of the mitochondrial electron transport chain	P21912
N-acetylated-alpha-linked acidic dipeptidase 2	*NAALAD2*	0.022	1.87	Dipeptidase activity	Q9Y3Q0
Aspartyl aminopeptidase	*DNPEP*	6.67 × 10^−6^	1.98	Protein metabolism	Q9ULA0
Replication protein A 14 kDa subunit	*RPA3*	0.003	2.02	Controls DNA repair and DNA damage checkpoint activation	P35244
Ubiquitin-40S ribosomal protein S27a	*RPS27A*	0.002	2.02	Postreplication repair	P62979
Ubiquitin-60S ribosomal protein L40	*UBA52*	0.002	2.02	Postreplication repair	P62987
Polyubiquitin-B	*UBB*	0.002	2.02	Postreplication repair	P0CG47
Polyubiquitin-C	*UBC*	0.002	2.02	Postreplication repair	P0CG48
Fatty aldehyde dehydrogenase	*ALDH3A2*	2.96 × 10^−5^	2.08	Catalyzes the oxidation of aliphatic aldehydes to fatty acids	P51648
Thioredoxin domain-containing protein 17	*TXNDC17*	3.91 × 10^−6^	2.19	Disulfide reductase with peroxidase activity	Q9BRA2
Glycine dehydrogenase (decarboxylating), mitochondrial	*GLDC*	2.17 × 10^−5^	2.23	Catalyzes the degradation of glycine	P23378
Delta-1-pyrroline-5-carboxylate dehydrogenase, mitochondrial	*ALDH4A1*	0.009	2.42	Involved in the interconnexion between the urea and tricarboxylic acid cycles	P30038
Catalase	*CAT*	5.92 × 10^−6^	2.66	Protection against oxidative stress	P04040
Proliferating cell nuclear antigen	*PCNA*	0.002	2.86	DNA replication and DNA repair	P12004
Tissue alpha-L-fucosidase	*FUCA1*	0.001	2.86	Related to glycoside metabolic process	P04066
Aldo-keto reductase family 1 member C3	*AKR1C3*	2.83 × 10^−5^	3.21	Oxidoreductase activity	P42330
Dipeptidyl peptidase 2	*DPP7*	1.24 × 10^−6^	3.40	Degradation of tripeptides	Q9UHL4
DNA ligase 3	*LIG3*	-	3-NT+EA	Correct defective DNA strand-break repair	P49916
Alcohol dehydrogenase 4	*ADH4*	-	3-NT+EA	Oxidoreductase activity	P08319
DNA-directed RNA polymerase II subunit RPB1	*POLR2A*	-	3-NT+EA	Transcription of DNA into RNA	P24928
Glutathione S-transferase Mu 3	*GSTM3*	-	3-NT+EA	Detoxification of endogenous compounds	P21266
Alcohol dehydrogenase 4	*ADH4*	-	3-NT+EA	Oxidoreductase activity, acting on the CH-OH group of donors, NAD or NADP as acceptor	A0A0D9SFB5

^1^ Fold change (FC) indicates the degree of quantity change for a particular protein between cells (3-NT vs. 3-NT+EA). FC <1 denotes a decrease in the concentration of protein; FC >1 indicates a significant increase in the concentration of the protein. When a given protein is only present in one of the groups, fold change cannot be measured, and such condition is denoted as “3-NT” (if protein is only present in 3-NT-exposed cells) or 3-NT+EA (if protein is only present in cells exposed to both chemical species). 3-NT3-NT3-NT3-NT.

## Data Availability

All data is contained within the article or supplementary material, including raw data from Proteomics.

## Supplementary Materials

The following supporting information can be downloaded at: https://www.mdpi.com/article/10.3390/antiox11122485/s1
