# Supplementary material for: Ellagic Acid Triggers the Necrosis of Differentiated Human Enterocytes Exposed to 3-Nitro-Tyrosine: An MS-Based Proteomic Study
Source: Antioxidants (Basel). 2022 Dec 17;11(12):2485. doi: 10.3390/antiox11122485 (PMC9774974; doi:10.3390/antiox11122485)
Supplement: Supplementary file 1 [file antioxidants-11-02485-s001.zip › manuscript.v9_SupplFigures.pdf]

Article

Ellagic Acid Triggers the Necrosis of Differentiated Human Enterocytes Exposed to 3-Nitro-Tyrosine: An MS-Based Proteomic Study

Silvia Díaz-Velasco [1], Josué Delgado [2], Fernando J. Peña [3] and Mario Estévez [1,*]

[1] Food Technology and Quality (TECAL), Institute of Meat and Meat Products (IPROCAR), Universidad de Extremadura, 10003, Cáceres, Spain
[2] Food Hygiene and Safety (HISEALI), Institute of Meat and Meat Products (IPROCAR), Universidad de Extremadura, 10003 Cáceres, Spain
[3] Spermatology Laboratory, Universidad de Extremadura, 10003 Cáceres, Spain
* Correspondence: mariovet@unex.es

Supplementary Materials

Citation: Díaz-Velasco, S.; Delgado, J.; Peña, F.J.; Estévez, M. Ellagic Acid Triggers the Necrosis of Differentiated Human Enterocytes Exposed to 3-Nitro-Tyrosine: An MS-Based Proteomic Study. *Antioxidants* **2022**, *11*, x. https://doi.org/10.3390/antiox11122485

Academic Editors: Giancarlo Aldini, Alessandra Altomare, Giovanna Baron

Received: 3 October 2022
Accepted: 9 December 2022
Published: 17 December 2022

Publisher's Note: MDPI stays neutral with regard to jurisdictional claims in published maps and institutional affiliations.

Copyright: © 2022 by the authors. Submitted for possible open access publication under the terms and conditions of the Creative Commons Attribution (CC BY) license (https://creativecommons.org/licenses/by/4.0/).

Supplementary Graphic (SG) 1

(A)

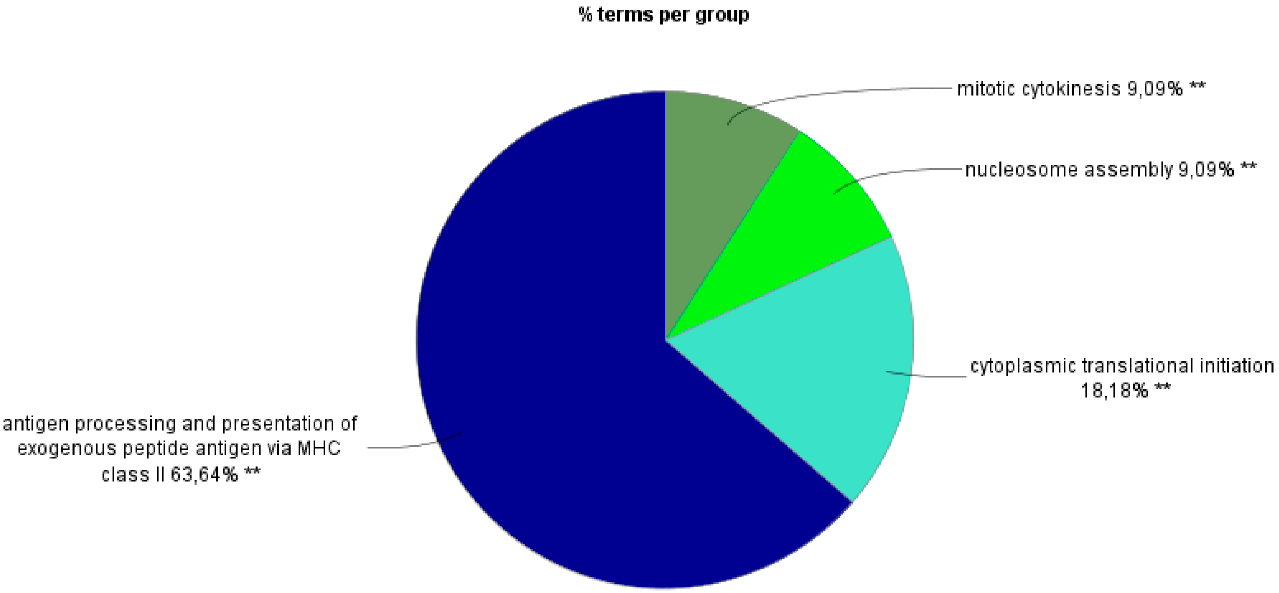

(B)

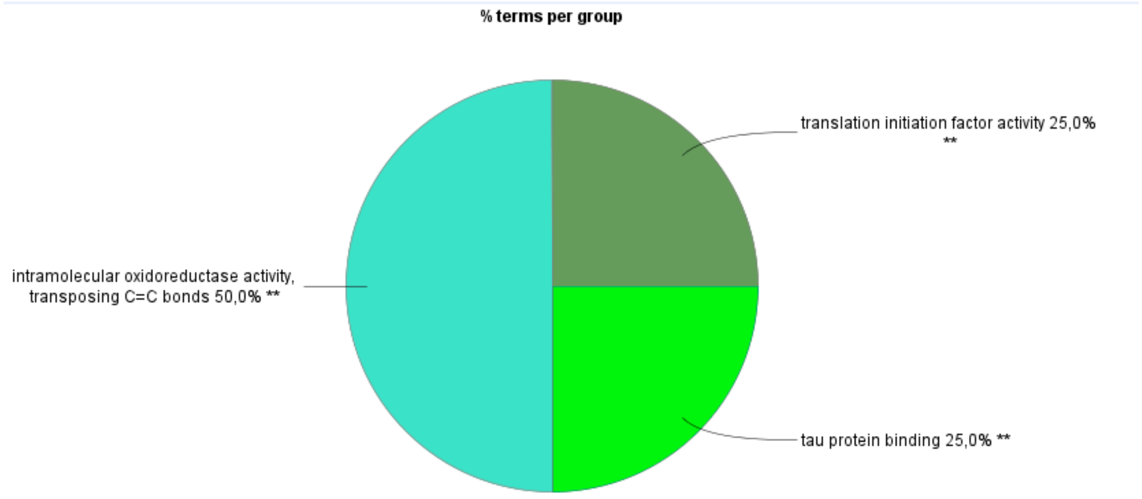

(C)

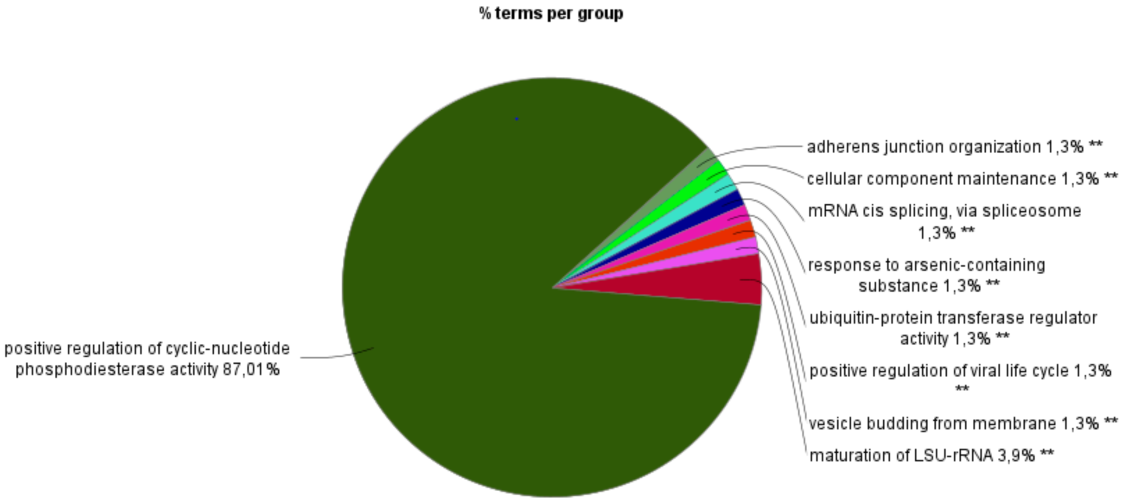

(D)

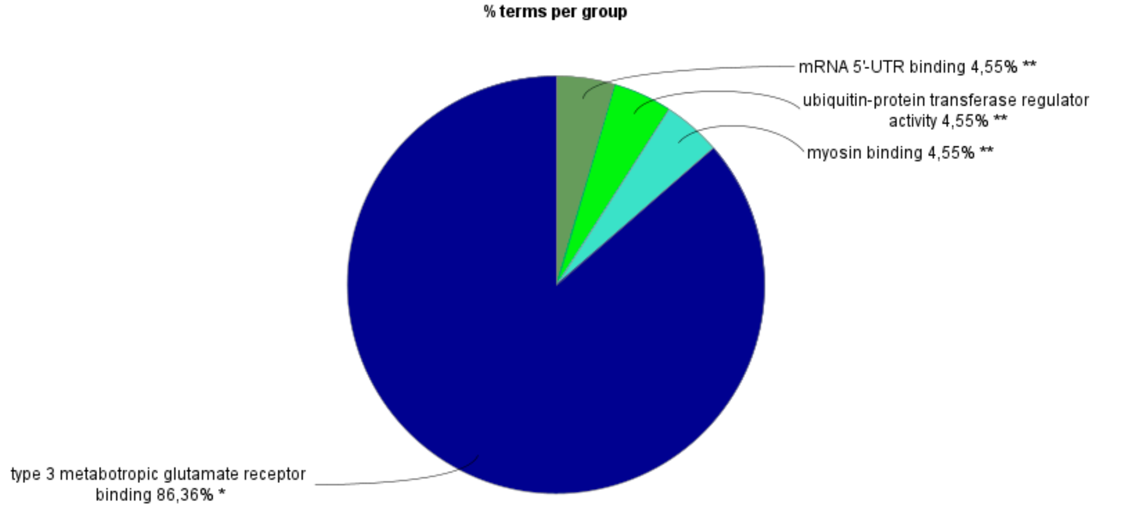

SG1. Percentages of proteins annotated by Gene Ontology (* $p < 0.05$, ** $p < 0.01$) found in lower relative quantity in biological processes (A) and molecular functions (B), and percentages of proteins found in higher relative quantity in biological processes (C) and molecular functions (D) on differentiated human enterocytes as affected by exposure to 200 μM 3-NT for 72 h.

Supplementary Graphic (SG) 2

(A)

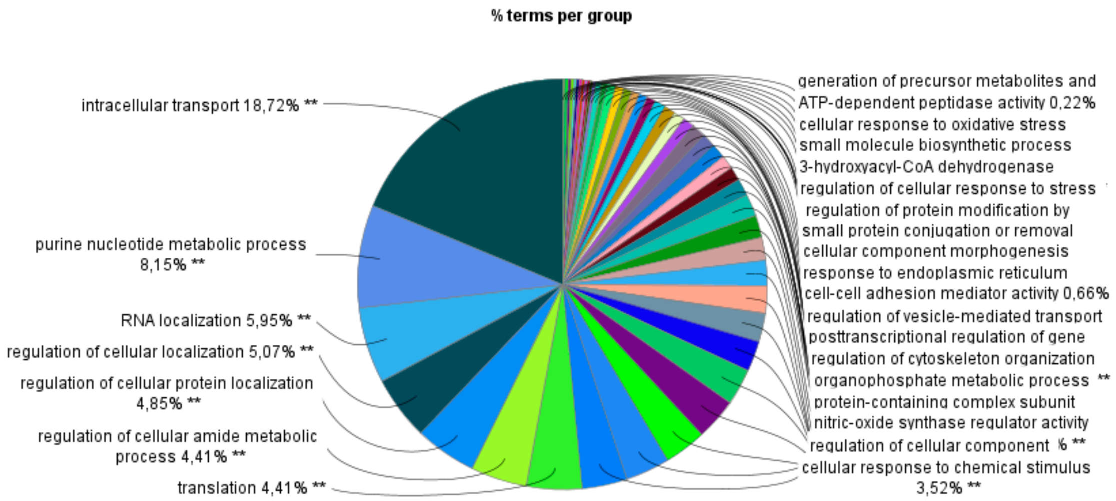

(B)

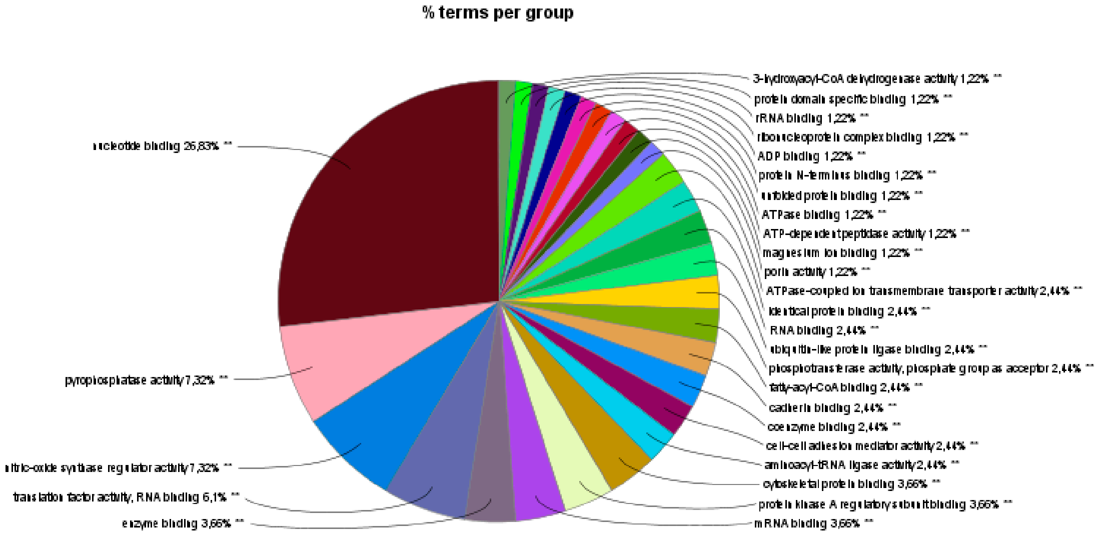

(C)

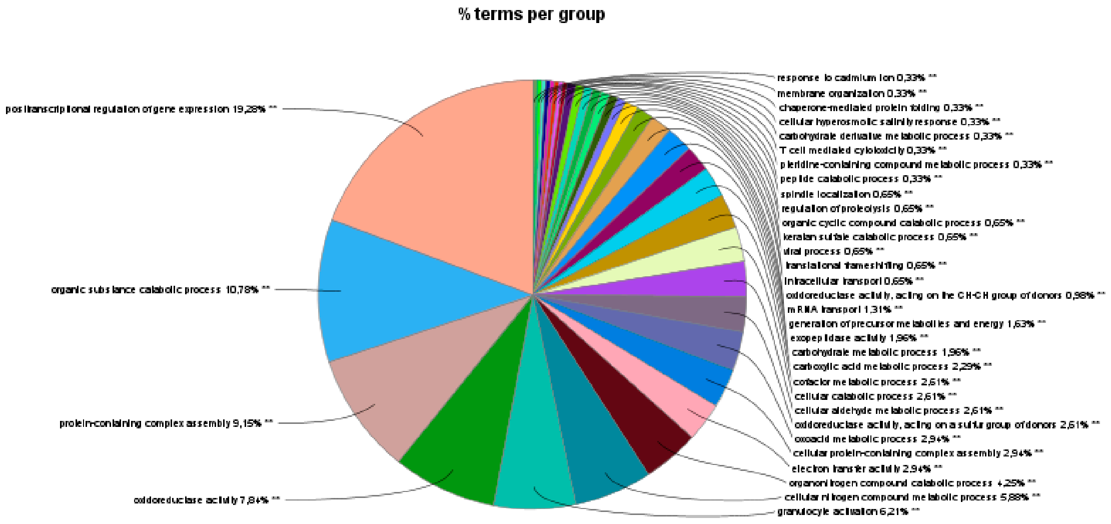

(D)

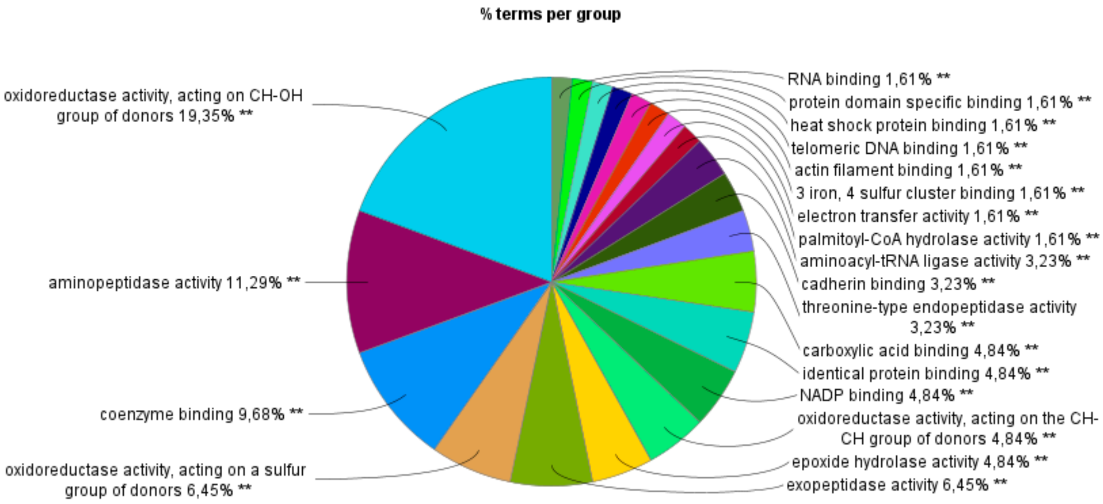

SG2. Percentages of proteins annotated by Gene Ontology (* $p < 0.05$, ** $p < 0.01$) found in lower relative quantity in biological processes (A) and molecular functions (B), and percentages of proteins found in higher relative quantity in biological processes (C) and molecular functions (D) on differentiated human enterocytes as affected by exposure to 200 μM 3-NT+EA for 72 h.

Supplementary Picture 1

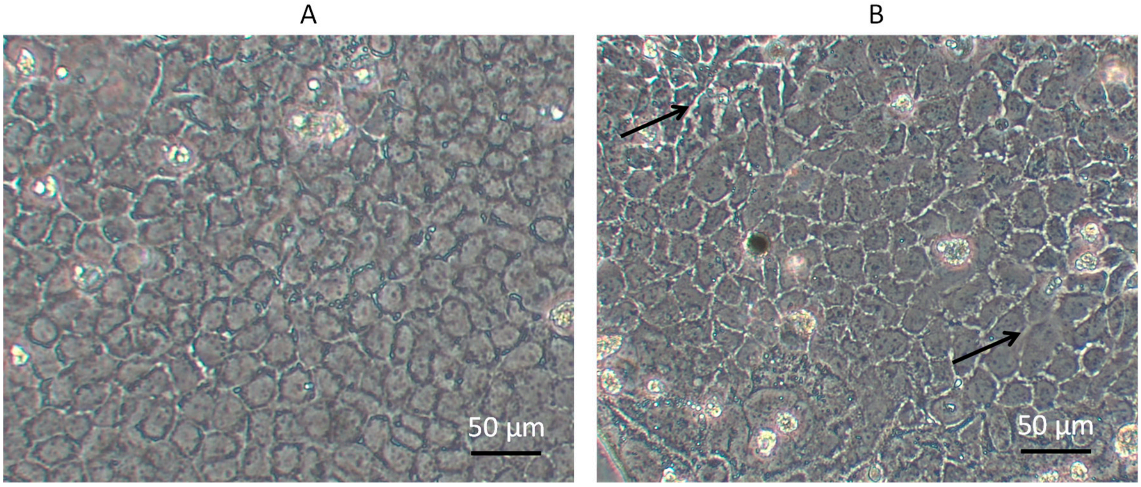

Picture 1. Differentiated CACO-2 cells growing in culture. (A) Cells growing at confluence under CONTROL conditions (37ºC/72 h) (B) Cells at confluence incubated in the presence of 3NT (200 µM) and EA (200 µM) (72 h). The treatment induced cell damage, which could be observed as cell lysis and the release of debris into the culture medium (marked by arrows). Scale bar represents 50 µm.
